# A selective c-Met and Trks inhibitor Indo5 suppresses hepatocellular carcinoma growth

**DOI:** 10.1186/s13046-019-1104-4

**Published:** 2019-03-18

**Authors:** Teng Luo, Shou-Guo Zhang, Ling-Fei Zhu, Fei-Xiang Zhang, Wei Li, Ke Zhao, Xiao-Xue Wen, Miao Yu, Yi-Qun Zhan, Hui Chen, Chang-Hui Ge, Hui-Ying Gao, Lin Wang, Xiao-Ming Yang, Chang-Yan Li

**Affiliations:** 1 State Key Laboratory of Proteomics, Beijing Proteome Research Center, National Center for Protein Sciences (Beijing), Beijing Institute of Lifeomics, Beijing, 102206 China; 2 Beijing Institute of Radiation Medicine, 27-Taiping Road, Beijing, 100850 People’s Republic of China; 3 An Hui Medical University, Hefei, 230032 China; 4 Guangdong pharmaceutical university, School of Pharmacy, Guangzhou, 510006 China; 5 School of Chemical Engineering and Technology, Department of pharmaceutical engineering, Tianjin University, Tianjin, 300072 China; 6 Institute of NBC Defence, Beijing, 102205 China

**Keywords:** Hepatocellular carcinoma, C-met, TrkB, Specific inhibitor, Therapeutic strategy

## Abstract

**Background:**

Human hepatocellular carcinoma (HCC) lacks effective curative therapy and there is an urgent need to develop a novel molecular-targeted therapy for HCC. Selective tyrosine kinase inhibitors have shown promise in treating cancers including HCC. Tyrosine kinases c-Met and Trks are potential therapeutic targets of HCC and strategies to interrupt c-Met and Trks cross-signaling may result in increased effects on HCC inhibition.

**Methods:**

The effects of Indo5 on c-Met and Trks activity were determined with in vitro kinase activity assay, cell-based signaling pathway activation, and kinases-driven cell transformation. The in vivo anti-tumor activity was determined with xenograft mice and liver orthotopic mice models. The co-expression of c-Met and TrkB in 180 pairs of HCC and adjacent normal tissues were detected using immunohistochemical staining.

**Results:**

Indo5, a novel lead compound displayed biochemical potency against both c-Met and Trks with selectivity over 13 human kinases. Indo5 abrogated HGF-induced c-Met signaling activation and BDNF/NGF-induced Trks signal activation, c-Met or TrkB-mediated cell transformation and migration. Furthermore, Indo5 significantly decreased the growth of HCC cells in xenograft mice and improved the survival of mice with liver orthotopic tumors. In addition, co-expression of c-Met and TrkB in HCC patients was a predictor of poor prognosis, and combined inhibition of c-Met and TrkB exerted a synergistic suppressive effect on HCC.

**Conclusions:**

These findings indicate that Indo5 is associated with marked suppression of c-Met and Trks co-expressing HCC, supporting its clinical development as an antitumor treatment for HCC patients with co-active c-Met and Trks signaling.

**Electronic supplementary material:**

The online version of this article (10.1186/s13046-019-1104-4) contains supplementary material, which is available to authorized users.

## Background

Selective tyrosine kinase inhibitors have shown promise in treating cancers driven by activated tyrosine kinases such as EGF receptor (EGFR) in non-small cell lung cancer (NSCLC), Bcr-Abl in chronic myelogenous leukemia (CML), and c-Kit in gastrointestinal stromal tumors (GIST) [1]. Sorafenib, a multikinase inhibitor that targets several serine/threonine and receptor tyrosine kinases including Raf, Vascular endothelial growth factor receptor (VEGFR), and platelet-derived growth factor receptors (PDGFR), is the current standard of care for patients with advanced hepatocellular carcinoma (HCC) [2, 3]. A pair of phase III studies indicated that sorafenib improved survival and the time to radiologic progression, leading to its approval for the treatment of advanced HCC [2]. However, it only extends the median life expectancy of patients by 1 year [2, 3]. Most patients eventually show disease progression, even if they are on a therapeutic regimen [4, 5]. Therefore, there is an urgent need to develop a novel molecular-targeted therapy for HCC.

Ongoing efforts to study hepatocarcinogenesis have identified an important role of c-Met signaling in the promotion of tumor growth, angiogenesis, and metastasis including HCC. c-Met transcription is increased in HCC tumors and overexpression c-Met receptor protein results in a poor prognosis [6]. In addition, other alterations such as genomic amplification, activating point mutations, inadequate degradation and receptor crosstalk also contribute to the progression and invasive growth of several malignancies including HCC [7]. In vitro studies also demonstrated the effects of HGF on phenotypical changes of HCC, including EMT, migration, and invasion [8]. In multiple HCC cell lines, c-Met knockdown decreases cell proliferation, colony formation, and migration in vitro, and suppresses tumor growth in vivo [9]. Moreover, the c-Met receptor has been known to be a key player in drug resistance [10]. In addition, c-Met also was reported to involve in regulation of the development of cancer stem cells in HCC via c-Met/FRA1/HEY1 cascade [11]. Therefore, c-Met is now regarded as one of the most promising therapeutic targets for the treatment of HCC. Different approaches have been described to interfere with the c-Met signaling pathway, such as antisense oligonucleotides, monoclonal antibodies, and specific c-Met inhibitors [7]. Currently, many clinical trials are being conducted for c-Met targeting in HCC management, using c-Met inhibitors such as INC280, foretinib, MSC2156119J, golvatinib, tivantinib, and cabozantinib [12]. Among these, tivantinib and cabozantinib are entering phase III randomized controlled trials. Although the use of c-Met inhibitors as a potentially viable treatment is supported by preclinical data, there are still concerns about the feasibility of utilizing c-Met targeting approaches. In particular, resistance and the side effects of taking c-Met inhibitors are issues that remain to be resolved.

Accumulated evidence have reported that aberrant c-Met activation can occur in various tumors through crosstalking with other receptors, including EGFR, chemokine receptor 4 (CXCR4), VEGFR-2 and FGFR [13]. Moreover, gene amplification of c-Met is observed to couple with enhancement of K-Ras oncogene [14], and the Wnt/β-catenin functional interaction with HGF/c-Met pathways in cancer cells is identified [15]. Importantly, preclinical data have demonstrated that these crosstalks play a key role in the growth and maintenance of the malignant phenotype and drug resistance, and thus combined blockade of the signaling pathways involved in these crosstalk achieve better treatment outcome in cancer.

The tropomyosin receptor kinase (Trk) family belongs to receptor tyrosine kinases and is composed of three homologous receptor tyrosine kinases: TrkA, TrkB, and TrkC, which specifically bind neurotrophins nerve growth factor (NGF), brain-derived neurotrophic factor (BDNF), and neurotrophin 4 (NT4) and neurotrophin 3 (NT3), respectively [16]. Neurotrophin and TrkA and TrkB receptors play an important role in the development and progression of various tumors [17]. For patients harboring alterations in Trks expression or activity, Trks inhibition emerges as an important therapeutic target, and multiple trials testing Trks-inhibiting compounds in various cancers are underway [18]. Recent studies demonstrated an association between Trk and HCC [19]. These studies reveal that a higher serum BDNF level was associated with a more advanced tumor status in the HCC patients [20]. Although NGF was negative in adult and developing livers, it was markedly elevated in focal hepatocytic lesions from early stages of carcinogenesis [21]. Moreover, TrkA and TrkB are demonstrated frequently overexpressed in HCC patients, and was significantly correlated with multiple and advanced stage of HCC [19]. Expressions of BDNF and TrkB were found in HCC cells, neutralizing antibody specific for BDNF or BDNF knockdown induced apoptosis and suppressed invasion of cells [22]. Importantly, lupeol, a naturally available triterpenoid with selective anti-cancerous, was reported to suppress HCC cell proliferation by inhibiting BDNF secretion [23]. Therefore, aiming at BDNF/TrkB signaling interruption may be an effective strategy to prevent HCC progression.

Recently, several lines of evidence have revealed a connection between c-Met and Trk receptors in promoting tumor progression in addition to their respective roles in the occurrence and development of tumors. Activated c-Met enhances TrkA signaling and the bioactivity of NGF [24], and TrkB up-regulates c-Met expression in neuroblastoma and enhances the invasion of tumor cells [25]. High expression of TrkA and c-Met and c-Met copy number gain/amplification were frequent events in salivary duct carcinoma (SDC), and high expression of TrkA and c-Met reveal the tendency to be related to poor prognosis in HER2-negative SDC [26]. Recent molecular studies revealed that both c-Met and Trk pathways transactivate each other in Glioblastoma (GBM) [27]. Moreover, several inhibitors such as K252a and Cabozantinib were show to inhibit kinases including c-Met and Trks, and suppresses the malignant phenotypes of many tumor cells including HCC [28]. Therefore, strategies to interrupt c-Met and Trk receptors cross-signaling may result in increased effects on tumor inhibition.

Here, we described Indo5 as a potent and selective c-Met and Trk inhibitor. Importantly, Indo5 exhibited the antitumor effects on HCC with in vitro models, in vivo xenograft, and orthotopic models. These findings supported its clinical development as an antitumor treatment for HCC patients with co-active c-Met and Trks signaling.

## Methods

### Reagents and cell culture

The human hepatocellular carcinoma cell lines HepG2, HCC LM3, MHCC97-L, MHCC97-H, BEL-7402, and SMMC-7721, human liver cell line L02, human neuroblastoma cell line SK-N-SH, human lung carcinoma cell lines A549 and NCI-H460, human fetal lung cell MRC-5, primary mice hepatocytes, and Madin-Darby Canine Kidney (MDCK) cell were all cultured in DMEM-H (Gibco) supplemented with 2 mM glutamine, 100 units/ml penicillin, 100 mg/ml streptomycin and 10% FBS. Murine pre-B cells (Ba/F3) were grown in RPMI 1640 containing 10% FBS and 10% WEHI-3 cell-conditioned medium as a source of murine IL-3. The compound 3-(1H-benzimidazol − 2-methylene)-5- (2-methylphenylaminosulfo)- 2-indolone (Indo5) was synthesized as previously described (US Patent No: 9642839B2). For oral gavage, Indo5 was dissolved in 0.5% CMC-Na.

### Cell growth assay

All cell growth assays were performed using the High Content Screening System (PerkinElmer). Briefly, 2 × 10^3^ cells were seeded in a 96-multiwell plate (CellCarrier-96, PerkinElmer). After 4 h, different concentrations of inhibitors were added to the wells. The plate was immediately put into the Operetta and incubated for 48 h at 37 °C and 5% CO_2_. Digital phase contrast images were sequentially acquired every 3 h using a 10 × objective with 6 fields per well. Image analysis was performed using the Harmony application to provide the algorithm.

### Mice

Female BALB/c athymic nude mice aged 5 to 6 weeks were purchased from Beijing Vital River Laboratory Animal Technology (Beijing Vital River Laboratory Animal Technology Co., Ltd., Beijing, China) and allowed to acclimate for 1 week. Animal care was in accordance with the institutional animal care guidelines. All animal experiments were approved by the Animal Ethics Committee of the Academy of Military Medical Science.

### Xenograft tumor model

First, 2 × 10^6^ HepG2 cells, 4 × 10^6^ MHCC 97H cells, 4 × 10^6^MHCC 97 L cells, 2 × 10^6^ A549 cells, or 5 × 10^6^ SMMC-7721 cells in a volume of 200 μl PBS were inoculated subcutaneously in the right flank of each mouse. Tumor growth was measured using vernier calipers, and tumor volume (mm^3^) was calculated as (L × W × H)/2. When the mean tumor volume reached 200 mm^3^, the mice were randomized into four groups and treatment was initiated. Indo5 in DMSO was injected intraperitoneally into the mice once daily at the indicated doses. Tumor volume were recorded every 3 days until the study was terminated. The mice were sacrificed after 21 days of treatment. Solid tumors were excised and weighed, and the inhibition rate was calculated as (Weight_Indo5 treatment group_-Weight_control group_)/Weight_control group_.

### Liver orthotopic xenograft mouse model

For the orthotopic xenograft mouse model, MHCC97H cells were inoculated subcutaneously in the right flank of each SCID mouse. When the tumor volumes reached approximately 1 cm^3^, the mice were sacrificed, and the tumors were isolated and cut into 1 mm^3^ pieces. The mice were anesthetized by an intraperitoneal injection of 1% sodium pentobarbital solution. A 1-cm longitudinal skin incision was made on the right upper axillary region of the abdomen of the mouse, the peritoneum was opened and the liver was exposed. The liver was punctured by a needle, and a 1 mm^3^ tumor piece was implanted. The liver was put back into the abdominal cavity gently, and the surgical opening was closed using a 5–0 surgical suture.

### Statistics

Data were expressed as the mean ± S.D. All statistical analyses were performed using the GraphPad Prism software. Statistical significance was calculated using a two-way ANOVA. A *p*-value of 0.05 or less was considered statistically significant.

## Results

### Indo5 inhibits the biological activities of HGF/c-met

**Fig. 1:**
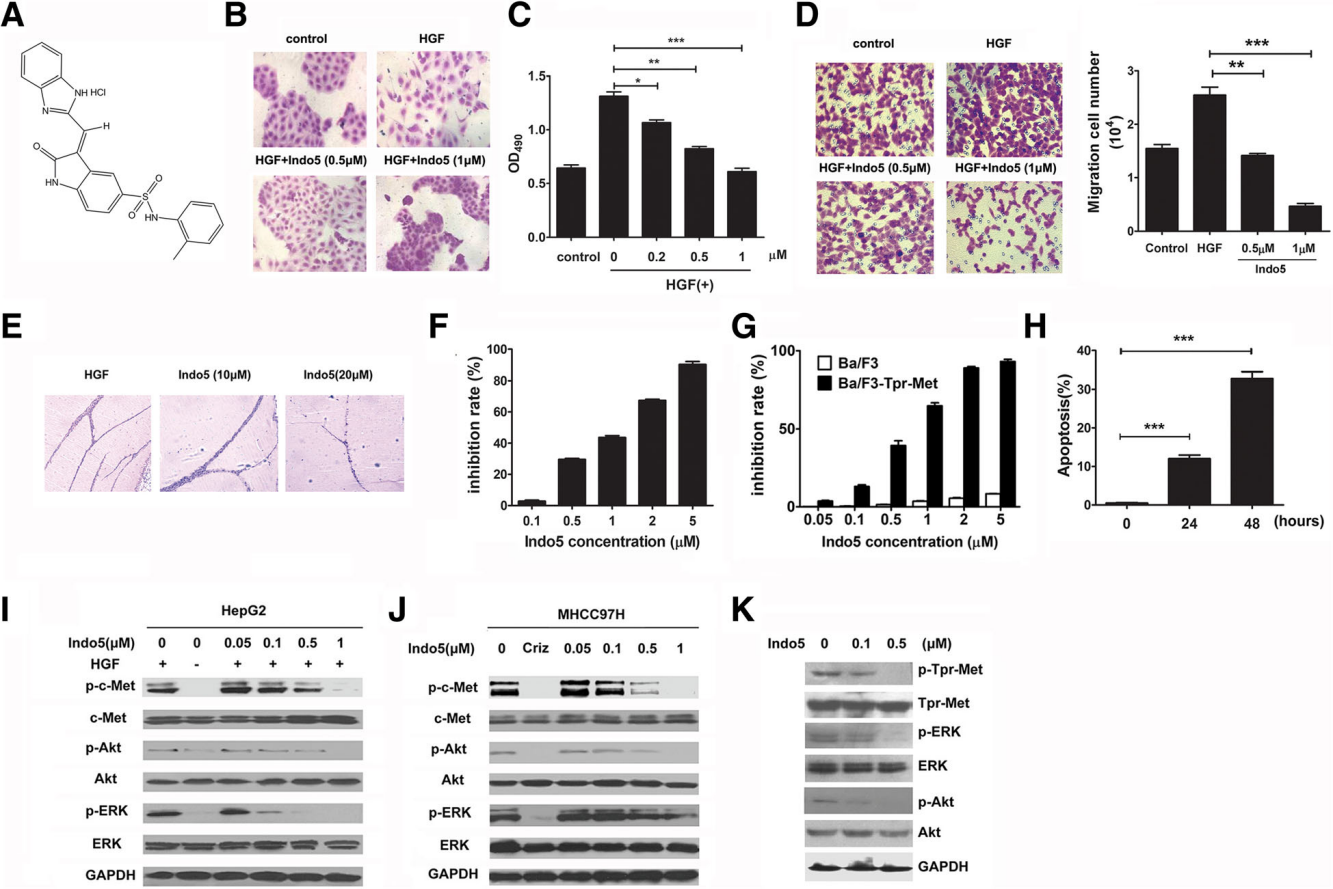
Indo5 inhibits the biological activities of HGF/c-Met and its downstream signaling pathways. (**a**) Structure of Indo5. (**b**) MDCK cells were pretreated with Indo5 for 2 h and then treated with HGF (20 ng/ml) for 12 h. The cell scattering was analyzed. (**c**) Mouse hepatocytes were pretreated with indicated doses of Indo5 for 2 h and then treated with HGF (20 ng/ml) for 48 h. Cell proliferation was analyzed with MTS assay. (**d**) HepG2 cells were pretreated with Indo5 for 2 h and then stimulated to migrate with 20 ng/ml HGF. The number of cells that migrated was measured 24 h later. (**e**) Matrigel plug assay with Indo5 treatment. (**f**) Effect of Indo5 on the growth of MHCC97H, Ba/F3-Tpr-Met and wild type Ba/F3 cells (**g**). Apoptosis of Ba/F3-Tpr-Met cells was analyzed with 0.5 μM Indo5 treatment at the indicated time (**h**). (I) HepG2 cells were pretreated with the indicated doses of Indo5 for 2 h and then treated with HGF (20 ng/ml) for 5 min. (**j**) MHCC97H cells were treated with different doses of Indo5 for 2 h. Crizotinib treatment (0.5 μM) was used as positive control. (**k**) Ba/F3-Tpr-Met cells were treated with the indicated doses of Indo5 for 2 h. Then, total cell lysates were prepared for Western blot using the indicated antibodies

3-(1H-benzimidazol-2-methylene)-5-(2-methylphenylaminosulfo)-2-indolone (Indo5) (Fig. 1a) was discovered as a highly potent inhibitor of HGF/Met signaling pathway in our previous assay using the cell-based screen in MDCK cells [29]. As shown in Fig. 1b, HGF-stimulated MDCK cell scattering was blocked by Indo5 in a dose-dependent manner. No obvious effect on the cell proliferation was observed (Additional file 1: Figure S1). To further address whether Indo5 would inhibit biological activities of HGF, we examined the ability of Indo5 to inhibit mitogenic activity of HGF. As shown in Fig. 1c, HGF stimulated proliferation of mouse hepatocytes and Indo5 dose-dependently inhibited HGF-induced cells proliferation. This inhibition by Indo5 was not due to a toxic effect of drug on the cells, since no cytotoxicity was observed following treatment of cells with 0.5 μM Indo5 even after 72 h treatment (data not shown). In addition, Indo5 treatment also significantly suppressed HGF-induced HepG2 cells migration in a dose-dependent manner (Fig. 1d) with a weak inhibitory effect on cell proliferation (Additional file 1: Figure S2). The effect of Indo5 on HGF-induced angiogenesis in vivo was also studied using a Matrigel plug assay and the result indicated that Indo5 dramatically blocked HGF-induced vasculature formation in vivo (Fig. 1e).

Human HCC cell line MHCC97H constitutively expresses activated c-Met and is sensitive to c-Met inhibitors. We showed that Indo5 exerted potent antiproliferative activity against MHCC97H (IC_50_ = 1.114 μM, Fig. 1f, Table 1). Tpr-Met is an oncogenic form of Met and stable transfection of Tpr-Met in Ba/F3 cells causes the malignant transformation of the cells to cytokine-independent growth [30]. To determine whether Indo5 reversed the oncogenic activity of Tpr-Met, Ba/F3-Tpr-Met cells were treated with Indo5 and the cell growth and apoptosis were measured. As shown Fig. 1g, Indo5 inhibited the IL-3-independent growth of Ba/F3-Tpr-Met cells in a dose-dependent manner, with IC_50_ value of 0.618 μM. In contrast, no obvious effect was observed on wild-type Ba/F3 cells proliferation after Indo5 treatment. Indo5 treatment also markedly induced cell apoptosis (Fig. 1h).

**Table 1:** Inhibition of cancer cell proliferation by Indo5

c-Met and TrkB expression levels	Cell line	IC_50_ (μM) ± SD
c-Met^hi^TrkB^hi^	HepG2	1.463 ± 0.015
	HCC-LM3	1.873 ± 0.012
	MHCC97H	1.114 ± 0.018
	MHCC97L	1.284 ± 0.016
	A549	1.62 ± 0.008
c-Met^lo^TrkB^hi^	SMMC-7721	3.598 ± 0.022
	SK-N-SH	2.982 ± 0.019
c-Met^lo^TrkB^lo^	NCI-H460	9.185 ± 0.035
	BEL-7402	5.605 ± 0.017
c-Met^lo^TrkB^lo^ (non-cancerous cells)	MRC-5	> 100
c-Met^hi^TrkB^lo^ (non-cancerous cells)	L02	32.524 ± 0.075

The indicated tumor cell lines were plated in 96-well culture plates for 24 h, treated for 72 h with DMSO or various concentrations (0.05 to 50 μM) of Indo 5. The experiment was repeated 3 times with 5 duplicates per group. Cell growth was measured by MTS assays. Dose response curves were generated for determination of IC _50_ for each cell line

### Indo5 suppresses HGF-induced c-met tyrosine phosphorylation and downstream signal pathways

Indo5 was identified as a potent c-Met kinase inhibitor with an IC_50_ value of 14.37 nM using an ELISA assay with recombinant c-Met kinase protein (Additional file 1: Table S1). To detected the effect of Indo5 on HGF-induced phosphorylation of c-Met and downstream signal pathways, we investigated the cellular kinase-targeting activity of Indo5 in human HCC cell lines (HepG2 and MHCC97H), and Ba/F3-Tpr-Met cell. As shown in Fig. 1i, Indo5 inhibited the HGF-induced c-Met signaling pathway activation in a dose-dependent manner. When the concentration reached to 0.5 μM, HGF-induced c-Met signaling pathway activation was dramatically suppressed. Moreover, exposure to Indo5 significantly inhibited c-Met phosphorylation in MHCC97H cells without HGF induction, with a complete abolishment at 1 μM (Fig. 1j). Consistently, this was accompanied by a dose-dependent inhibition of phosphorylation of downstream signaling molecules, Akt and ERK1/2. Similar results were recapitulated in Ba/F3-Tpr-Met cells (Fig. 1k). The IC_50_ value of Indo5 inhibition c-Met is 14.37 nM in vitro, but in cellular kinase-targeting activity assay, the Indo5 concentration required for c-Met inhibition is over 0.1 μM. We speculated that high concentration needed for c-Met inhibition in cell culture might be due to the instability of Indo5, actually, the half-life of Indo5 is 5 min in plasma (data not shown). Analysis of the time course of Indo 5 on the c-Met phosphorylation suggested that the inhibitory effect of Indo 5 was lasts from 2 h to 6 h (Additional file 1: Figure S3).

### Indo5 is a potent inhibitor of Trk kinases

**Fig. 2:**
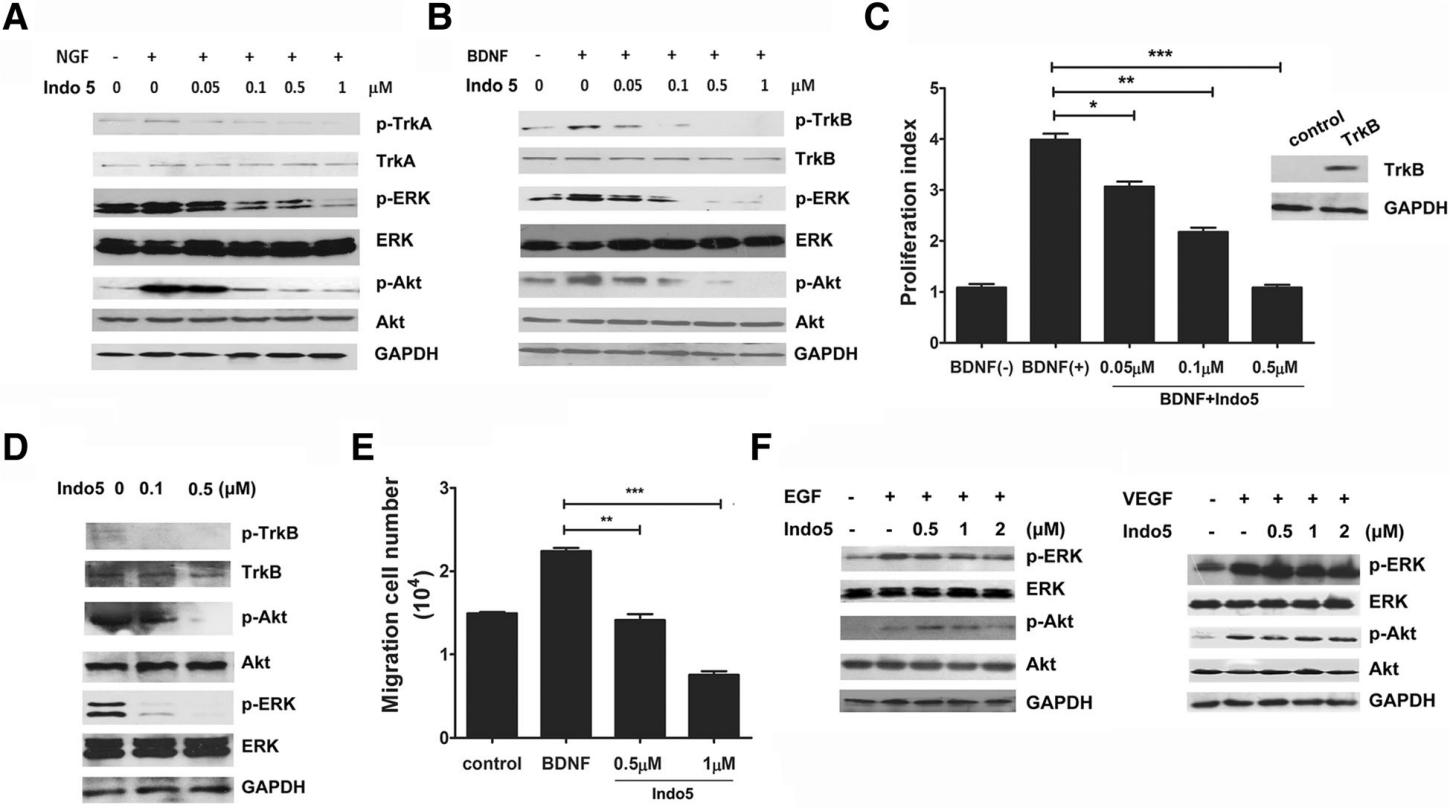
Indo5 is a potent inhibitor for Trk kinase. (**a**) SK-N-SH cells were pretreated with the indicated doses of Indo5 for 2 h and then treated with NGF (500 ng/ml) for 5 min. HepG2 cells were pretreated with Indo5 for 2 h and then treated with BDNF (50 ng/ml) for 5 min (**b**). Total cell lysates were prepared for Western blot analysis with the indicated antibodies. (**c**) Ba/F3-TrkB cells were pretreated with Indo5 for 2 h and then treated with BDNF (50 ng/ml) for 48 h. Cell proliferation was measured and the proliferation index was calculated. The expression of TrkB in Ba/F3-TrkB cells was confirmed by Western blot (right panel). The inhibitory effect of Indo5 on the BDNF-induced TrkB signaling pathway was investigated (**d**). (**e**) HepG2 cells were pretreated with Indo5 for 2 h followed by BDNF (50 ng/ml) treatment for 24 h. The number of cells that migrated was measured 24 h later. Data are shown as the mean ± S.D. and are representative of three independent experiments. ** *p* < 0.01, *** *p* < 0.001. (**f**) A549 cells were pretreated with Indo5 for 2 h and then treated with 100 ng/ml EGF (left) or 100 ng/ml VEGF (right) for 5 min. Cell lysates were prepared for Western blot

To investigate whether Indo5 was specifically against c-Met, Indo5 was profiled against a panel of 15 human kinases by an in vitro kinase activity assay. Unexpectedly, Indo5 was found to inhibit the kinase activities of TrkA, and TrkB with IC_50_ values of 28 nM, and 25 nM, respectively (Table S1). No significant effect of Indo5 was observed on other 13 tyrosine kinases. These data suggested that Indo5 may be a potent inhibitor for Trk kinases. To confirm, we investigated the effect of Indo5 on the Trk signaling pathway in SK-N-SH cells. The data suggested that Indo5 pre-treatment inhibited NGF-induced signaling in a dose-dependent manner, and TrkA signaling was almost completely inhibited at a concentration of 1 μM Indo5 (Fig. 2a). Moreover, Indo5 pretreatment decreased BDNF-induced TrkB signaling in a dose-dependent manner in HepG2 cells, which express high level of TrkB (Fig. 2b).

A previous study reported that upon BDNF stimulation, TrkB transfected Ba/F3 cells had an accelerated growth rate compared with mock transfectants [31]. We constructed Ba/F3 cells stably transfected with TrkB and evaluated the effect of Indo5 on BDNF-induced cell growth. As shown in Fig. 2c, Indo5 treatment inhibited the BDNF-induced growth of TrkB-transfected cells in a dose-dependent manner. The inhibitory effect of Indo5 on BDNF-induced TrkB activation was confirmed by Western blot (Fig. 2d). BDNF-induced HepG2 cell migration was also suppressed with Indo5 treatment (Fig. 2e).

We also investigated the effects of Indo5 on the EGF and VEGF receptor-mediated signaling pathway and the result suggested that Indo5 treatment had no effect on the activation of the signaling pathway (Fig. 2f). These data confirmed the specificity of Indo5 for the c-Met and Trk signaling pathways.

### Indo5 effectively inhibits cell growth of c-met and Trk coexpressing HCC cells

**Fig. 3:**
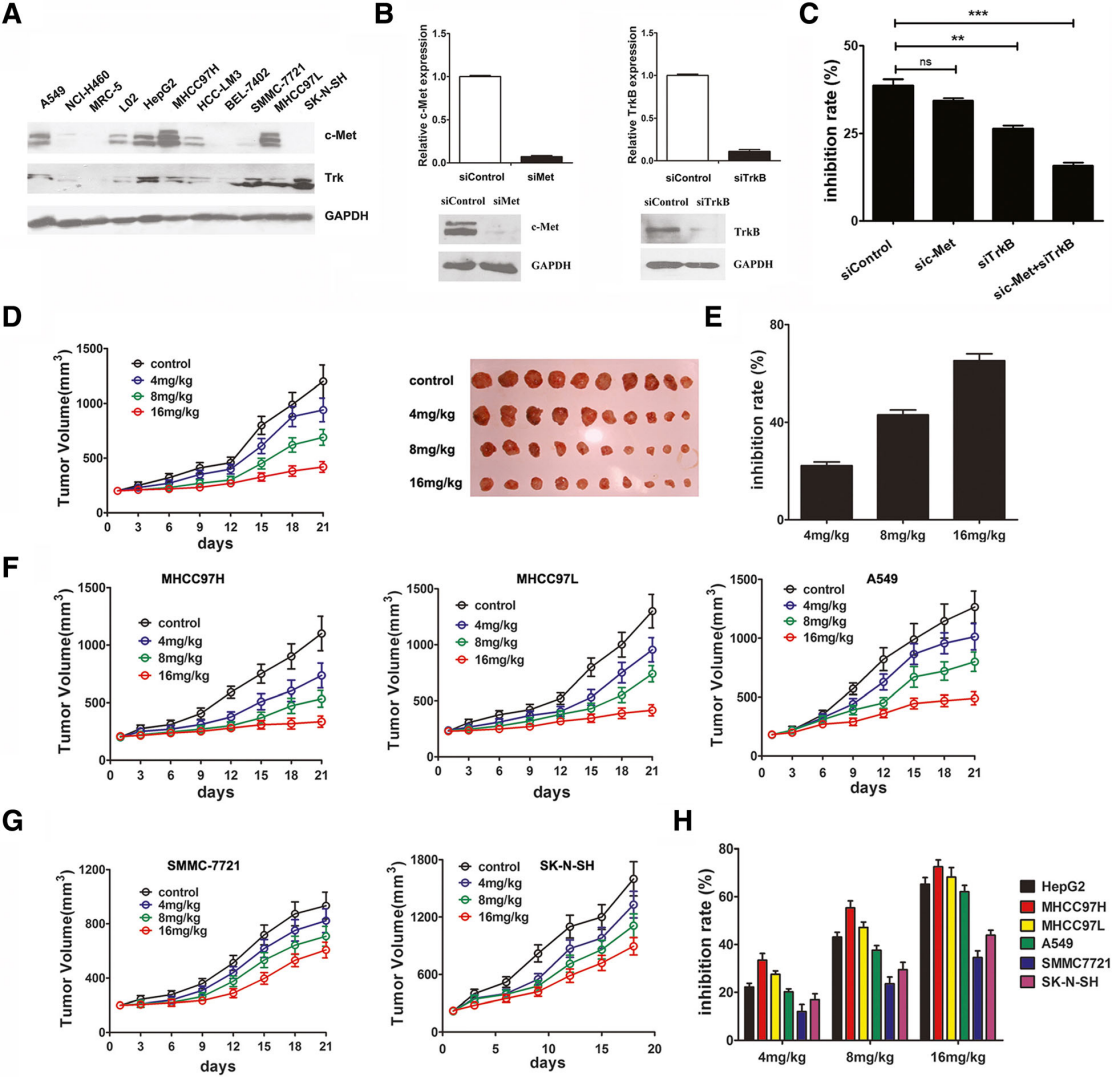
Indo5 effectively inhibits c-Met and Trk coexpressing HCC cells growth in vitro and in vivo. (**a**) The expression profile of c-Met and Trk in the indicated cell lines. (**b**) HepG2 cells were transfected with either a scrambled control, c-Met siRNA, or TrkB siRNA. The expression levels of c-Met and TrkB were analyzed by real-time PCR analysis (upper panel) and Western blot (below panel). (**c**) HepG2 cells transfected with specific siRNA or control siRNA were treated with Indo5 (1 μM) for 48 h and cells growth was measured. The inhibition rate was calculated. (**d**) Indo5 inhibited the growth of HepG2 cell line tumor xenografts in nude mice (left panel). Images of tumors are shown in the right panel. The inhibition rate of Indo5 on HepG2 cell tumor xenograft growth was calculated according to the weight of tumors (**e**). (**f**) The effect of Indo5 on the growth of c-Met/TrkB co-expressing cell lines (MHCC97H, MHCC97L and A549) and other cells lines (SMMC-7721 and BEL-7402) (**g**) tumor xenografts in nude mice. The inhibition rates of Indo5 on the indicated cell line tumor xenografts were calculated (**h**)

We detected the expression profile of c-Met and TrkB in several human tumor cells line. As Fig. 3a shows, high level of c-Met and TrkB were found coexpressed in human HCC cell lines HepG2, HCC-LM3, MHCC97H, MHCC97L, and human lung carcinoma cell lines A549 cells, only TrkB expression is present in human HCC cell line SMMC-7721 and human neuroblastoma cell line SK-N-SH cells.

We further investigated the effect of Indo5 on the growth of these cell lines. Indo5 significantly inhibited cell growth in a dose-dependent manner in c-Met and TrkB coexpressing cell lines (c-Met^hi^TrkB^hi^), the IC_50_ value of Indo5 ranged from 1.114 ± 0.018 μM to 1.873 ± 0.012 μM. In cells expressing TrkB alone or both low (c-Met^lo^TrkB^hi^ and c-Met^lo^TrkB^lo^), the IC_50_ value is much higher, ranging from 2.982 ± 0.019 μM to 9.185 ± 0.035 μM (Table 1, Additional file 1: Figure S4). Interestingly, Indo5 treatment exerted little anti-growth effect in non-cancerous cells (cytotoxicity IC_50_ values of > 30 μM) even in L02 cells the expression levels of c-Met and TrkB were high. These results indicated that the inhibition effect of Indo5 on HCC growth was closely correlated with the expression levels of c-Met and Trk.

To further confirm the role of c-Met and Trk in Indo5-induced HCC cell growth inhibition, specific siRNA oligonucleotides were used to knock down c-Met and Trk in HepG2 cells (Fig. 3b) and the effect of Indo5 on cell growth was detected. As Fig. 3c shows, combined knockdown of c-Met and Trk attenuated dramatically the inhibition effect of Indo5, suggesting that Indo5 suppresses the growth of HCC cells depending on the presence of c-Met and Trk.

### Indo5 exhibits anti-tumor activity in the xenograft mouse model

We also tested the in vivo anti-tumor activity of Indo5 by using the HepG2 xenograft nude mice model. As described, a significant number of HepG2 cells were injected subcutaneously into the right flanks of nude mice, and xenograft tumors were established. The administration of Indo5 dramatically inhibited HepG2 xenograft growth in nude mice (Fig. 3d). Notably, Indo5 exerted a dose-dependent effect in vivo; at a 16 mg/kg dose, the Indo5 inhibition rate was 65.2% (Fig. 3e). Furthermore, increased apoptosis (TUNEL staining) was observed in Indo5-treated tumor tissues (Additional file 1: Figure S5). The mouse body weights were almost unchanged between the groups, indicating that the administration of Indo5 was generally safe to the experimental mice **(**Additional file 1: Figure S6**)**. Thus, we showed that Indo5, at well-tolerated doses, suppressed HepG2 xenograft growth in nude mice. Similar results were obtained in MHCC97H, MHCC97L, and A549 xenograft mice (Fig. 3f) and no obvious effect of Indo 5 on body weight was observed in these mice models (data not shown).

We further measured the anti-tumor activity of Indo5 in cell lines expressing TrkB or c-Met alone. In SMMC-7721 and SK-N-SH xenograft mice, although a dose-dependent inhibitory effect was detected (Fig. 3g), the activity of Indo5 was not as desirable as that in cells expressing high levels of c-Met and TrkB (Fig. 3h).

These results indicated a selective and potent anti-proliferative activity by Indo5 against c-Met and TrkB coexpressing cells, especially HCC cells.

### Indo5 demonstrates anti-tumor activity and improves overall survival in a liver orthotopic mouse model

**Fig. 4:**
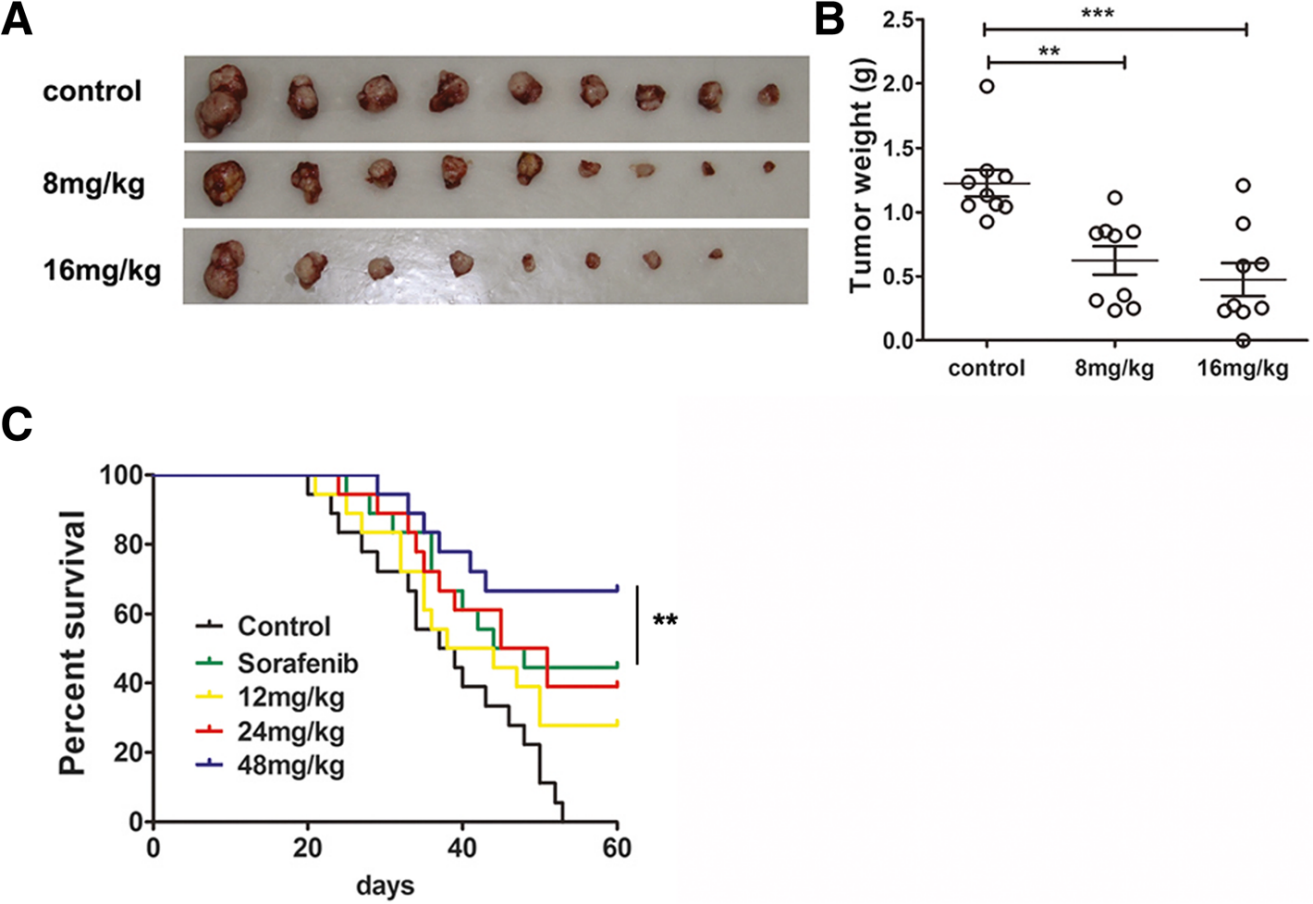
Indo5 demonstrates anti-tumor activity and improves overall survival in a liver orthotopic mouse model. (**a**) The effect of Indo5 on the growth of orthotopic HepG2 cell xenografts. Mice bearing tumor xenografts were intraperitoneally injected with the indicated doses of Indo5 once daily for 3 weeks. Representative vehicle- and Indo5-treated HepG2 tumors are shown. The tumor weights were measured (**b**). (**c**) Therapeutic effects of Indo5 on survival of mice bearing MHCC97H orthotopic tumors. Mice bearing MHCC97H orthotopic tumors were administered with the indicated doses of Indo5 or 50 mg/kg Sorafenib once daily by oral gavage. Tumor-bearing mice were sacrificed when they became moribund. Kaplan–Meier survival analysis is shown

An in vivo orthotopic mouse model of HepG2 cells was used to demonstrate the anti-tumor effect of Indo5. As shown in Fig. 4a and b, Indo5 treatment showed a markedly dose-dependent inhibitory effect on the intrahepatic implanted tumors. At an 8 mg/kg dose of Indo5, the rate of tumor growth inhibition was approximately 49%, and at a 16 mg/kg dose, the inhibition rate increased to 61.2%. In addition, the overall health of the animals was not adversely affected by the Indo5 treatment compared to control (data not shown).

We further investigated the overall survival of Indo5-treated orthotopic mice. Sorafenib, the first-line HCC therapy drug, was used as the positive control. A Kaplan–Meier survival analysis confirmed that in the control group all the mice died within 53 days, whereas mice injected with Sorafenib showed a 44% survival rate (Fig. 4c). The survival rate of mice treated with 24 mg/kg Indo5 was 40%, which was comparable to that in the Sorafenib-treated group. Interestingly, in mice treated with 48 mg/kg Indo5, the overall survival rate was 77.7%, which is significantly higher than that of the Sorafenib-treated group. Consistent with the above results, no obvious weight changes were observed with Indo5 treatment (Additional file 1: Figure S7). Histological examination of the liver tissue from vehicle-treated mice revealed the presence of large areas of proliferating tumor cells in the tumor tissues. In contrast, in Indo5 treated mice the tumor area was much smaller, no obvious tumor cells were observed in mice treated with 48 mg/kg Indo5 (Additional file 1: Figure S8). Moreover, significant tumor metastasis in control group was observed in lung and Indo 5 treatment led to dramatic decrease of HCC metastasis in lung (Additional file 1: Figure S9). These results showed that Indo5 has anti-tumor activity and improved the overall survival in liver orthotopic mouse model.

### Combined targeting of c-met and TrkB kinases is a therapeutic strategy in HCC

**Fig. 5:**
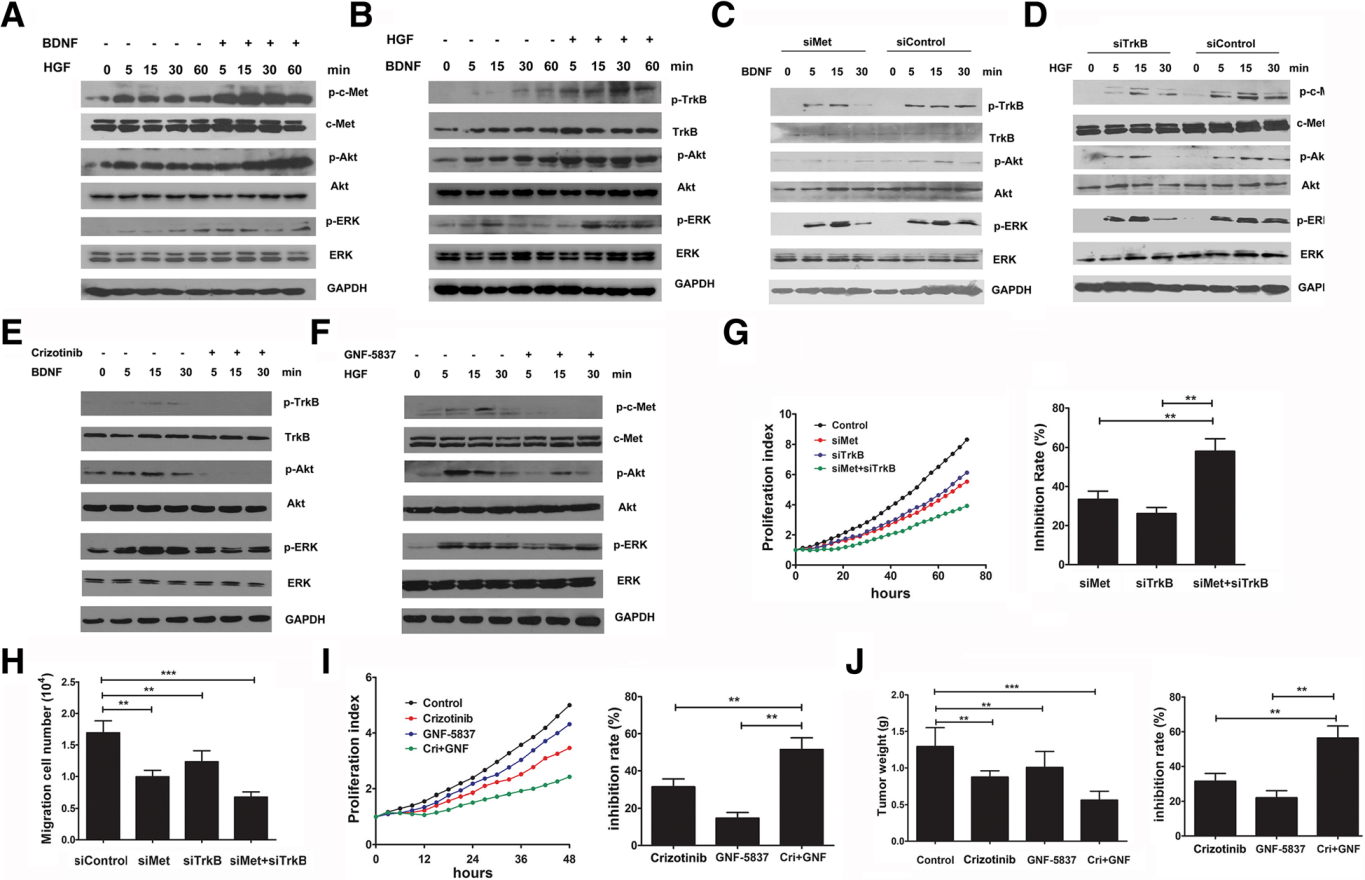
Combined targeting of c-Met and TrkB kinases is a therapeutic strategy in HCC. (**a**) HepG2 cells were treated with HGF (10 ng/ml) alone or HGF (10 ng/ml) and BDNF (10 ng/ml) for the indicated time and the activation of c-Met signaling pathway was examined. (**b**) HepG2 cells were treated with BDNF (10 ng/ml) alone or BDNF (10 ng/ml) and HGF (10 ng/ml) for the indicated time and the activation of TrkB signaling pathway was examined. (**c**) HepG2 cells were transfected with c-Met siRNA or TrkB siRNA oligonucleotides (**d**) for 48 h and then treated with BDNF (50 ng/ml) or HGF (20 ng/ml) for the indicated time. The activation of the TrkB and c-Met signaling pathways were examined. (**e**) HepG2 cells were pre-treated with Crizotinib (0.5 μM) or GNF-5837 (4 μM) (**f**) for 2 h and then stimulated with BDNF (50 ng/ml) or HGF (20 ng/ml) for the indicated time. The activation of the TrkB and c-Met signaling pathways were examined. (**g**) HepG2 cells were transfected with the indicated siRNA oligonucleotides and cell growth was measured within 72 h (left panel). The inhibition rate of siRNA oligonucleotides was calculated according to the cell number at 72 h (right panel). (**h**) HepG2 cells were transfected with the indicated siRNA oligonucleotides for 48 h, and the cell migration activity was examined within 24 h. (**i**) HepG2 cells were treated with Crizotinib (100 nM) or GNF-5837 (2 μM), as indicated, and the cell growth was measured within 48 h (left panel). The inhibition rate was calculated (right panel). (**j**) Mice bearing HepG2 cell tumor xenografts were intraperitoneally injected with Crizotinib (16 mg/kg) or GNF-5837 (16 mg/kg) as indicated for 3 weeks. The tumor weights were measured, and the inhibition rate was calculated. Data are shown as the mean ± S.D. and are representative of three independent experiments. **p < 0.01, ***p < 0.001

Since c-Met and TrkB was coexpressing in HCC, we hypothesized that a cooperation between c-Met and TrkB pathways might be present in HCC. To test this hypothesis, HepG2 cells were co-treated with HGF and BDNF and the activation of c-Met and TrkB were detected. As shown in Fig. 5a, low dose of HGF treatment induced c-Met phosphorylation and downstream signaling pathway activation, and combined application of BDNF significantly augmented HGF-induced phosphorylation of c-Met, ERK, and Akt. Similarly, HGF cotreatment also elevated the activation of TrkB and downstream signaling pathways induced by BDNF (Fig. 5b).

We further investigated the connection between c-Met and TrkB signaling pathways using specific siRNA oligonucleotides or inhibitors. As Fig. 5c shows, although knockdown of c-Met did not affect the expression level of TrkB, a significant attenuation of BDNF-induced TrkB phosphorylation and downstream signaling pathway activation was observed (Additional file 1: Figure S10). Similarly, TrkB knockdown also blocked HGF-induced c-Met phosphorylation and activation of downstream pathways (Fig. 5D, Additional file 1: Figure S11) without affecting the expression level of c-Met. Crizotinib (also known as PF-2341066) is a multi-kinase inhibitor with known action against c-Met, anaplastic lymphoma kinase (ALK) and c-ros oncogene 1 (ROS1). GNF-5837 is a potent, selective inhibitor of TrkA, TrkB and TrkC [32]. HepG2 cells were pre-treated with Crizotinib or GNF-5837 for 2 h and then stimulated with BDNF or HGF, the activation of TrkB and c-Met signaling pathways were detected. As shown in Fig. 5e, c-Met kinase activity inhibition led to a significant attenuation of BDNF-induced TrkB signaling pathway activation (Additional file 1: Figure S12). Similarly, TrkB kinase activity inhibition also suppressed the HGF-induced c-Met signaling pathway activation (Fig. 5f, Additional file 1: Figure S13). These results indicated that c-Met and TrkB pathways present cooperation relationship in HCC.

To investigate the role of the cooperation between c-Met and TrkB signaling in HCC, we used specific siRNA oligonucleotides to knockdown the expression of c-Met and TrkB in HepG2 cells and measured cell growth for 72 h. As shown in Fig. 5g, knockdown of c-Met or TrkB alone inhibited cell growth and their combined knockdown led to the synergistic inhibition of cell growth in HepG2 cells. Similar results were obtained in MHCC97H cells (Additional file 1: Figure S14). A Transwell migration assay indicated that the combined knockdown of c-Met and TrkB decreased the migration activity of HCC more significantly than down-regulating one kinase alone (Fig. 5h). Furthermore, when cells were treated with HGF or BDNF at low dose, the migration activity was increased weakly. However, combined application of HGF and BDNF significantly enhanced the migration in HepG2 cells (Additional file 1: Figure S15).

We further investigated the effects of Crizotinib and GNF-5837 on HCC growth. As Fig. 5i shows, the inhibition rate in cells treated with 100 nM Crizotinib was 30%, and in cells treated with 2 μM GNF-5837, the growth inhibition rate was only 14%. However, the growth inhibition rate was much more higher when the two inhibitors were combined (inhibition rate is 52%).

We also investigated the effect of the combination of Crizotinib and GNF-5837 on the growth of HCC cells in vivo. As shown in Fig. 5j, the inhibition rate was 28% in the Crizotinib treatment alone group and 21% in the GNF-5837 treatment alone group. However, in the combined treatment group, the inhibition rate increased to 56%.

Taken together, our results indicated that combined inhibition of c-Met and Trk kinase is more effectively in suppressing the growth of HCC cells with coexpression of c-Met and Trk than inhibition one kinase alone.

### Co-upexpression of c-met and TrkB in HCC patients predicts poor prognosis

**Fig. 6:**
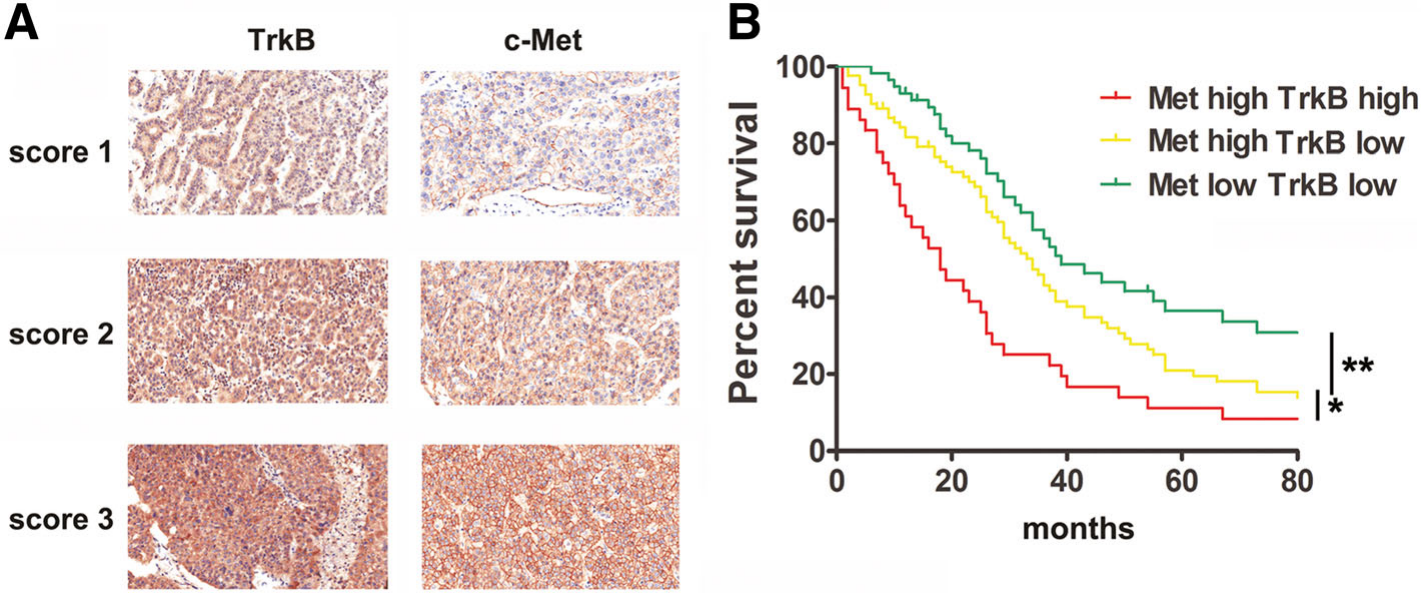
Co-upexpression of c-Met and TrkB in HCC patients predicts poor prognosis. (**a**) Representative images of different immunohistochemistry staining scores of c-Met and TrkB in HCC tissues. (**b**) Coexpression of c-Met and TrkB in HCC patients predicted poor prognosis

To future investigate the coexpression of c-Met and TrkB in human HCC tissues, c-Met and TrkB expression in the 180 pairs of HCC and adjacent normal tissues is determined using immunohistochemical (IHC) staining with specific c-Met and TrkB antibodies. Figure 6a depicts representative photomicrographs of the IHC analysis. Among the 180 cases, upexpression of c-Met and TrkB was noted in 119 (66%) and 40 (22%) patients compared to adjacent normal tissues, respectively; co-upexpression of c-Met and TrkB was observed in 36 (20%) of HCC tissues. In tumor tissues of TrkB up-regulated expression patients, 90% (36/40) patients showed a co-upexpression of c-Met expression (Additional file 1: Figure S9, Table S2). These results indicated that c-Met upexpression in HCC was correlated with TrkB expression.

To further evaluate the significant contribution of c-Met and TrkB coexpression in the prognosis of patients with HCC, 180 patients with HCC were divided into four groups according to the c-Met and TrkB expression levels in their tumors: c-Met ^high^ TrkB ^low^ (*n* = 83), c-Met ^high^ TrkB ^high^ (*n* = 36), c-Met ^low^ TrkB ^low^ (*n* = 57), and c-Met ^low^ TrkB ^high^ (*n* = 4). Since the patient number in the last group was too small for statistical analysis, we analyzed the overall survival in the other three groups. As shown in Fig. 6b, patients in c-Met ^high^ TrkB ^low^ group exhibited poor overall survival (OS) compared to patients in c-Met ^low^ TrkB ^low^ (median OS times were 35 and 39 months, respectively; difference = 4 months, *p* = 0.0344), and the group c-Met ^high^ TrkB ^high^had shorter OS times than did the group c-Met ^high^ TrkB ^low^ (median OS times were 18 and 34 months, respectively; difference = 16 months, *p* = 0.0015). These data indicate that the co-upregulation of c-Met and TrkB may be a potential factor for predicting the prognosis of HCC.

## Discussion

In this report, we identify Indo5, a novel indolinone derivative, as a tyrosine kinase inhibitor that targets c-Met and Trks in vitro and in vivo. Indo5 potently inhibited proliferation and survival of Tpr-Met and TrkB transfected Ba/F3 cells. Moreover, HCC cell lines with high c-Met and Trks coexpression were more sensitive to Indo5 and inhibition of c-Met and TrkB significantly reduced the antitumor activity of Indo5. These results suggested that the antitumor activity of Indo5 in HCC is mediated by suppression of oncogenic signaling of c-Met and TrkB. Of special interest was the observation that Indo5 treatment improved the survival of mice with liver orthotopic model generated by MHCC97H cells, which expresses high levels of c-Met and TrkB, with Indo5 showing similar or superior activity to Sorafenib. In addition, we showed that co-expression of c-Met and TrkB in HCC patients was a predictor of poor prognosis, and combined inhibition of c-Met and TrkB exerted a synergistic suppressive effect on HCC. These findings indicate that Indo5 is associated with marked suppression of c-Met and Trks co-expressing HCC, supporting its clinical development as an antitumor treatment for HCC patients with co-active c-Met and Trks signaling.

Indo5(3-(1H-benzimidazol-2-methylene)-5-(2-methylphenylaminosulfo)-2-indolone) contains the indolone core structure, which have been identified as a versatile scaffold for the development of protein kinase inhibitors [33]. By altering the substituents on the pyrrole and oxindole rings, it is possible to tune the potency and selectivity of the compounds for inhibition of various kinases such as SU5416, SU11274, SU11248 and SU6597 [34]. Indo5 selectively inhibited the kinase activities of c-Met, TrkA, and TrkB with IC_50_ values of 14.37 nM, 28 nM, and 25 nM, respectively. Furthermore, we noticed that while Indo5 displayed similar IC_50_s against the kinase activity of TrkA and TrkB in vitro (Table S1), it seemed that TrkB was more sensitive to TrkA in cells. This differential activity of Indo 5 on the signaling mediated by TrkA and TrkB might due to a different response to NGF and BDNF of SK-N-SH and HepG2 cells. No significant effect of Indo5 was observed on other tyrosine kinases including EGFR, VEGFR, FGFR1 and c-Met family member, Ron. Although the specificity of Indo5 kinase inhibition still requires further studies, our findings provide evidence that Indo5 exerts a potent cytotoxicity toward subsets of HCC cells characterized by high c-Met and Trk co-expression levels. In the development of tyrosine kinase inhibitors, a balance between toxicity and efficacy must be achieved. In many cases, the ideal anticancer effect can be achieved by inhibiting multiple kinases, which in turn increases its toxicity. In our experiment, while sorafenib treatment induced up to 10% body weight loss, and Indo5 treatment was not associated with loss of body weight even at the highest dose as single agent (48 mg/kg), indicating Indo5 was well tolerated in mice.

The role of HGF/c-Met and BDNF/TrkB signaling is reported in several cancers including HCC. Potential crosstalk between these signaling pathways is described in neuroblastoma [25], salivary duct carcinoma (SDC) [26], and Glioblastoma (GBM) [27]. The crosstalk between c-Met and Trks pathways were recently reported with mechanistic explanations in glioblastoma [27]. In this article, the authors showed that c-Met and Trk signaling transactivate to each other, and targeting both pathways simultaneously results in more efficient pathway suppression. Our present study indicate that combined application of HGF and BDNF significantly augmented signaling activation induced by each other, suggesting that the crosstalk between c-Met and Trks pathways is present in HCC cells. Interestingly, we found that inhibition of c-Met by specific siRNA or inhibitor did not downregulate TrkB expression in protein levels, but significantly impairs BDNF- induced TrkB signal activation. Conversely, downregulation of TrkB signaling significantly suppresses HGF-induced c-Met activation without affecting c-Met expression. These data indicate that there may be a mechanism of crosstalk between c-Met and TrkB in HCC different from that in GBM. It has been reported that c-Met plays an important role in activating or potentiating the response of other RTKs or vice versa through protein-protein interaction including EGFR, IGFR and RET [35]. c-Met cooperates with EGFR and plays a key role in drug resistance. EGFR inhibitors can block HGF-mediated proliferation and motility [36], and HGF stimulation could induce EGFR phosphorylation [35], pointing out the reciprocal regulation of both receptors. Therefore, detection of interaction between c-Met and Trk receptors may help to understand the precise mechanism of their crosstalk in HCC.

Combination targeting c-Met and other receptors exhibited a better anti-tumor effect than single agent administration. We showed that combined inhibition of c-Met and TrkB with siRNA or specific inhibitors exerted a synergistic suppressive effect on growth and migration of HCC cells, indicating that combined targeting of c-Met and Trk kinases is a useful therapeutic strategy. We demonstrate that co-expression of c-Met and TrkB is present in about 20% HCC patients and association with poor prognosis, indicating that co-expression of c-Met and TrkB may be a biomarker for HCC personalized therapy. Base on our above studies, we speculate that in a subclass of HCC patients with co-upexpression of c-Met and TrkB might benefit from targeted therapy with c-Met and Trk inhibitors. In addition to Indo5, several compounds have been shown to suppress c-Met and Trks such as K252a [28], Cabozantinib [37] and Merestinib [38]. Among them, antitumor effects of cabozantinib and K252a were examined in cultured HCC cells as well as in vivo models. Cabozantinib is an orally bioavailable small molecule inhibitor of c-Met, RET, ROS1, Alk, VEGFR2, and TRK receptors with approved uses for the treatment of metastatic medullary thyroid cancer, prostate cancer and advanced renal cell carcinoma [39]. However, these inhibitors are nonselective inhibitors with targeting multikinase and it is debatable whether an agent with such broad-spectrum activity is optimal. For example, K252a, is an inhibitor of the Trks, the PDGF receptor, and c-Met. Cabozantinib is a nonselective tyrosine kinase inhibitor with activity against c-Met, VEGFR2, FLT3, KIT, AXL, and RET [40]. Merestinib is a potent inhibitor of NTRK, it also targets additional kinases such as the TAM receptors (AXL, MERTK, and TYRO3), and MKNK1 and MKNK2, which may also contribute to anti-tumor growth [38]. Our present study suggested that Indo 5 is a selective inhibitor of c-Met and Trks without affecting the activity of other kinases such as PDGFR, EGFR, AXL, FGFR, IGF1R, FLT1 etc., which might indicate relative low toxicity of Indo 5.

## Conclusions

We have identified Indo5 as a novel inhibitor targeting c-Met and Trks. Our results show that Indo5 suppresses the growth of HCC cells with high levels of c-Met and Trk, providing a potentially new and practical pharmacological approach for treating HCC patients with co-active c-Met and Trks signaling.

## Additional file

Additional file 1: A selective c-Met and Trks inhibitor Indo5 suppresses hepatocellular carcinoma growth. (DOC 20 kb)

## Abbreviations

BDNF: Brain-derived neurotrophic factor
c-Met: c-mesenchymal-epithelial transition factor-1
HCC: Hepatocellular carcinoma
HGF: Hepatocyte growth factor
Indo5: 3-(1H-benzimidazol-2-methylene)-5-(2-methylphenylaminosulfo)-2-indolone EGF Epidermal growth factor
NGF: Nerve growth factor
Trk: Tropomyosin-related kinase
VEGF: Vascular endothelial growth factor
