# Supplementary material for: A selective c-Met and Trks inhibitor Indo5 suppresses hepatocellular carcinoma growth
Source: J Exp Clin Cancer Res. 2019 Mar 18;38:130. doi: 10.1186/s13046-019-1104-4 (PMC6421704; doi:10.1186/s13046-019-1104-4)
Supplement: Supplementary file 1 — A selective c-Met and Trks inhibitor Indo5 suppresses hepatocellular carcinoma growth. (DOC 20 kb) [file 13046_2019_1104_MOESM1_ESM.doc]

**Additional file**

**A selective c-Met and Trks inhibitor Indo5 suppresses hepatocellular carcinoma growth**

Teng Luo, Shou-Guo Zhang, Ling-Fei Zhu, Fei-Xiang Zhang, Wei Li, Ke Zhao, Xiao-Xue Wen, Miao Yu, Yi-Qun Zhan, Hui Chen, Chang-Hui Ge, Hui-Ying Gao, Lin Wang, Xiao-Ming Yang, Chang-Yan Li

**Summary**

The supplementary information (SI) file includes: supplementary material and methods, 2 supplementary tables, and 16 supplementary Figures.

**Supplementary** **Materials and** **Methods**

**Reagents and antibodies**

Crizotinib and GNF-5837 were purchased from Sigma-Aldrich Co. LLC (PZ0191-5MG,SML0844-5MG). HGF, NGF, and BDNF were purchased from  PeproTech, Inc.(100-39, 450-01, 450-02). The primary antibodies, c-Met, p-Met (Tyr1234/1235) , p-Trk (TrkA(Tyr490)/TrkB(Tyr516)), Akt, and p-Akt (Ser473), were all purchased from Cell Signaling Technology (MA, USA). Trk antibody was purchased from AbClonal Biotech Co. (Oxfordshire, UK). ERK-, p-ERK(Thr202/Tyr204), HRP-conjugated secondary antibodies were purchase from Santa Cruz (Santa Cruz).

**Primary mice hepatocytes isolation**

Primary mice hepatocytes were isolated by a two-step collagenase perfusion and cultured in DMEM containing 10% FBS, 2 mM L -glutamine, 1 mM sodium pyruvate and antibiotics .

**Western blot analysis**

For western blots, cells were lysed with M-PER® Mammalian Protein Extraction Reagent (Pierce, Rockford, IL, USA). Then, western blot analysis was performed according to standard procedures. Chemiluminescent detection was conducted using supersignal substrate (Pierce) according to the manufacturer’s specifications.

**RNAi**

The small interfering RNA (siRNA) oligos of c-Met , TrkB and scramble control were purchased fromSanta Cruz Biotechnology. siRNAs were transfected into cells using Lipofectamine 2000 (Invitrogen) at a concentration of 20 nM.

**MTS assay**

Cells were seeded in each well of 96-well plates and left overnight to adhere. Absorbance was determined by using the CellTiter 96 Aqueous One Solution Reagent (Promega) according to the manufacturer’s protocol. All experiments were conducted three times.

**In vitro kinase activity assay**

The activity of Indo5 was screened against a protein kinase panel of 15 human protein kinases by HD biosciences using the HDB Kinase Selectivity Profiling Services (HD biosciences Corporation, Shanghai, China). Protocols are available at http://www.hdbiosciences.com/EngKinase.htm. The activity of c-Kit, c-Kit (V654A) and EphB1 kinases were analyzed using Caliper assay format, The activity of other twelve kinases were analyzed using Kinase Glo Plus assay format. The activity of c-Met was analyzed using CycLex® Met Kinase Assay/Inhibitor Screening Kit INCLUDEPICTURE "http://ruo.mbl.co.jp/bio/images/ico_recommend.png" \* MERGEFORMATINET

(MBL life science).

**Migration assay**

For the migration assay, 2×10^5^ cells were seeded on 8μm polycarbonate membrane (Transwell; Costar). After 8h, cells were treated with different concentrations of Indo5, while in the bottom medium, HGF or BDNF was added. After 24h migration cells were counted.

**Cell scattering assay**

Cells were plated into 96-well plates (1.5 × 10^3^ cells/well) and grown until small colonies appeared. The serum was removed and cells were then stimulated with 100ng/ml HGF in the presence of Indo5. After 24h incubation, cells were fixed with 90% methanol for 30min at room temperature and stained by 1% crystal violet. Scattered clones were photographed.

**Matrigel Plug assay**

6-week old female BALB/c mice were subcutaneously injected with 700μL of Matrigel containing HGF(50ng/ml) with or without Indo5(10μM, 20μM). After 7 days, mice were sacrificed and the Matrigel plugs were fixed in 10% buffered formaldehyde, which was embedded in paraffin, and sectioned. The sections were stained with hematoxylin and eosin(H&E). H&E staining was performed to identify the formation and infiltration of new, functional microvessles.

**Tissue microarrays and immunohistochemical staining**

Tissue microarray chips that contained 180 cases of paired HCC tumor and peritumor specimens (HLiv-HCC180Sur-03, HLiv-HCC180Sur-04) were purchased from Shanghai Outdo Biotech Company (Shanghai, China). Ethical approval for this study was given by the Medical Ethics Committee of Shanghai, China. All specimens spotted on the tissue microarray chips included complete postoperative follow-up information for 2–3.7 years. Tumor staging was evaluated according to the tumor node metastasis classification of malignant tumors. Antibodies against human c-Met (1:500, Cell signaling Technology) or TrkB (1:100, Cell signaling Technology) were used for immunohistochemistry according the manufacture’s instructions. All slides were examined and scored by two pathologists who were blinded to clinical patient data. Each specimen was assigned a score according to the staining intensity score (no staining = 0; weak staining = 1, moderate staining = 2, strong staining = 3) and the extent of stained cells (0% = 0, 1–24% = 1, 25–49% = 2, 50–74% = 3, 75–100% = 4). The final immunoreactive score was determined by multiplying the intensity score with the extent of score of stained cells, ranging from 0 (the minimum score) to 12 (the maximum score).

**Supplementary Tables**

**Table** S**1 IC_50_ value of Indo5 on activities of different kinases *in vitro***

| Kinases | IC_50_ (nM) |
| --- | --- |
| TrkA | 28 |
| TrkB | 25 |
| c-Met | 14.37 |
| PDGFRα | >10000 |
| FGFR1 | >10000 |
| AXL | >10000 |
| FLT1 | >10000 |
| EPHA1 | >10000 |
| IGF1R | >10000 |
| EGFR | >10000 |
| InsR | >10000 |
| C-KIT | >10000 |
| C-KIT(v654A) | >10000 |
| Ephb1 | >10000 |
| RET | >10000 |
| EGFR(T790,L858R) | >10000 |

**Table S2 Expression level of c-Met and TrkB in HCC patients**

| Expression | TrkB high | TrkB low | Total |
| --- | --- | --- | --- |
| c-Met high | 36 | 83 | 119 |
| c-Met low | 4 | 57 | 61 |
| Total | 40 | 140 | 180 |

**Supplementary Figures**

**
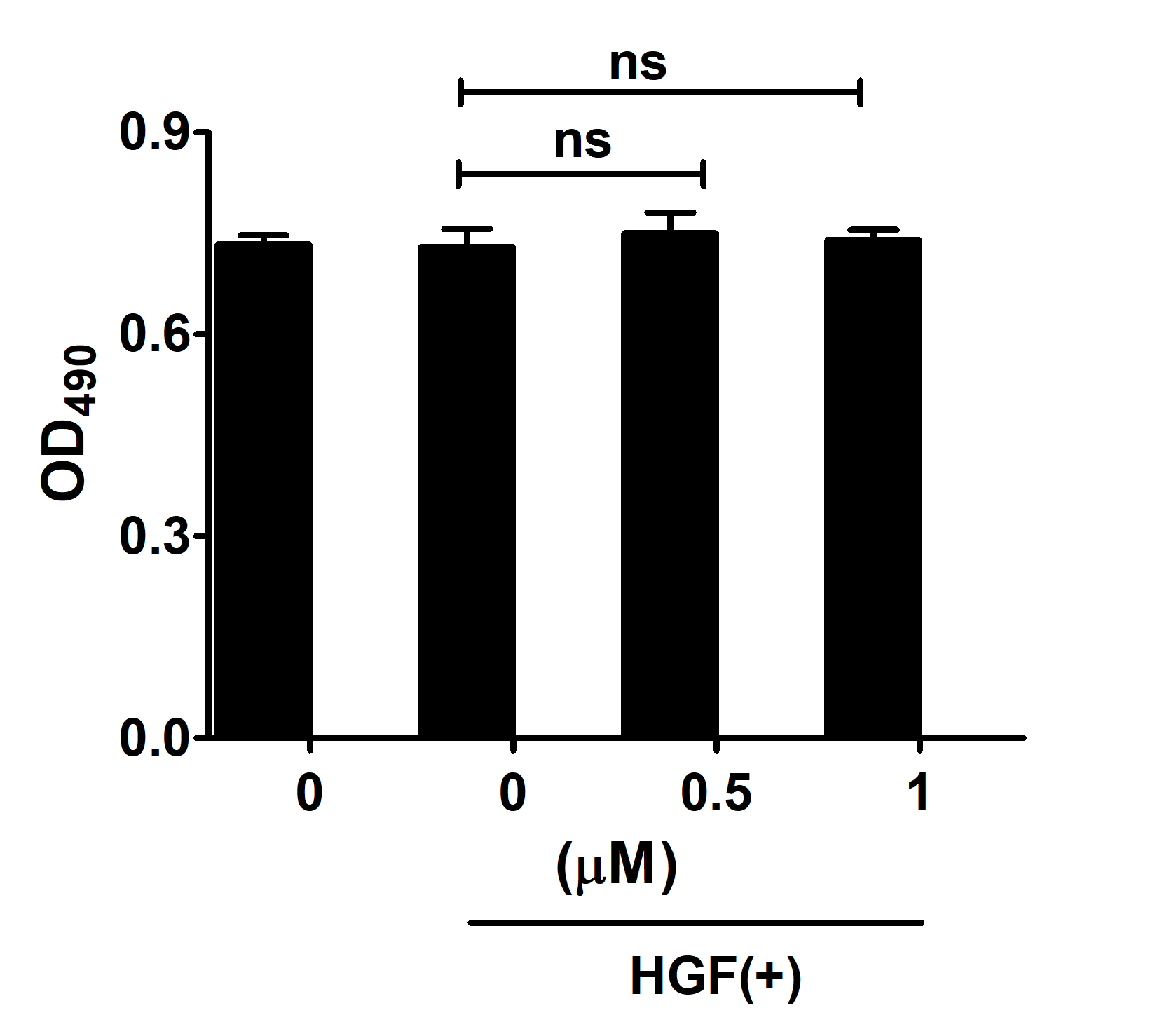
**

**Figure S1.** MDCK cells were pretreated with Indo5 for 2 h and then treated with HGF (20 ng/ml) for 12 h. Cell proliferation was anaylzed with MTS assay.

**
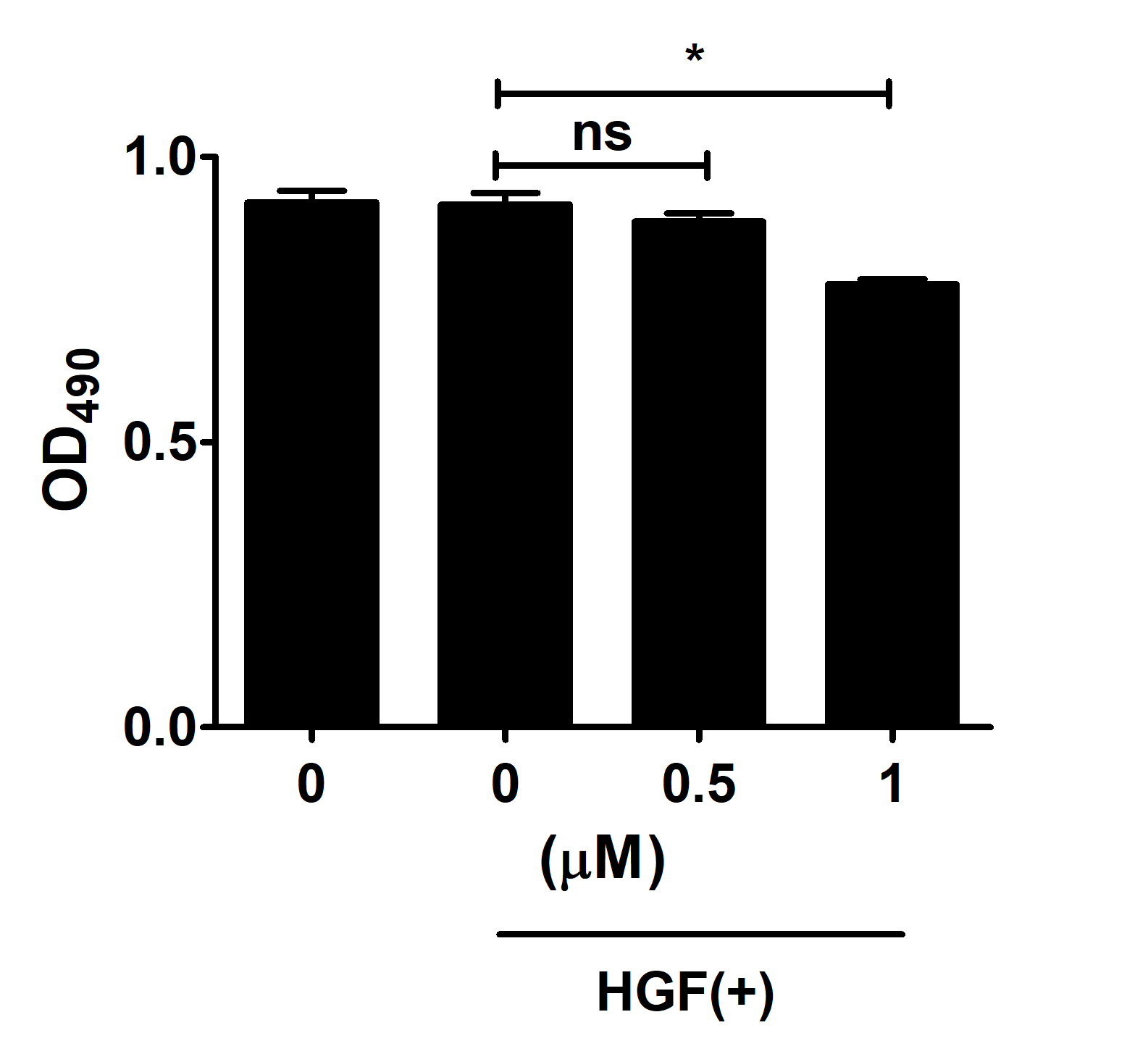
**

**Figure S2.** HepG2 cells were pretreated with Indo5 for 2 h and then stimulated to migrate with 20 ng/ml HGF. Cell proliferation was anaylzed with MTS assay.

**
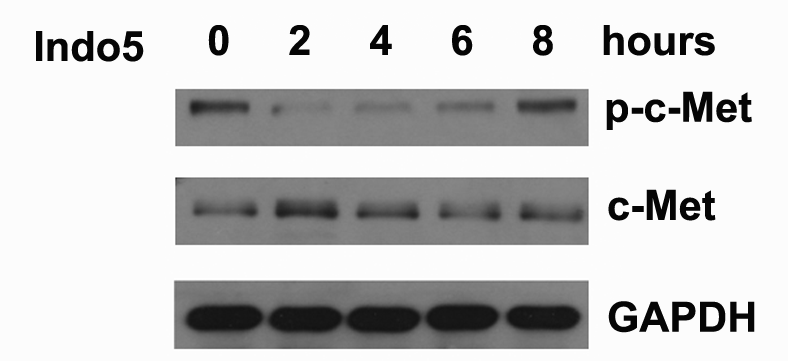
**

**Figure S3.** MHCC97H cells were treated with 0.5μM Indo5 for the indicated time and the phosphorylation of c-Met was investigated.


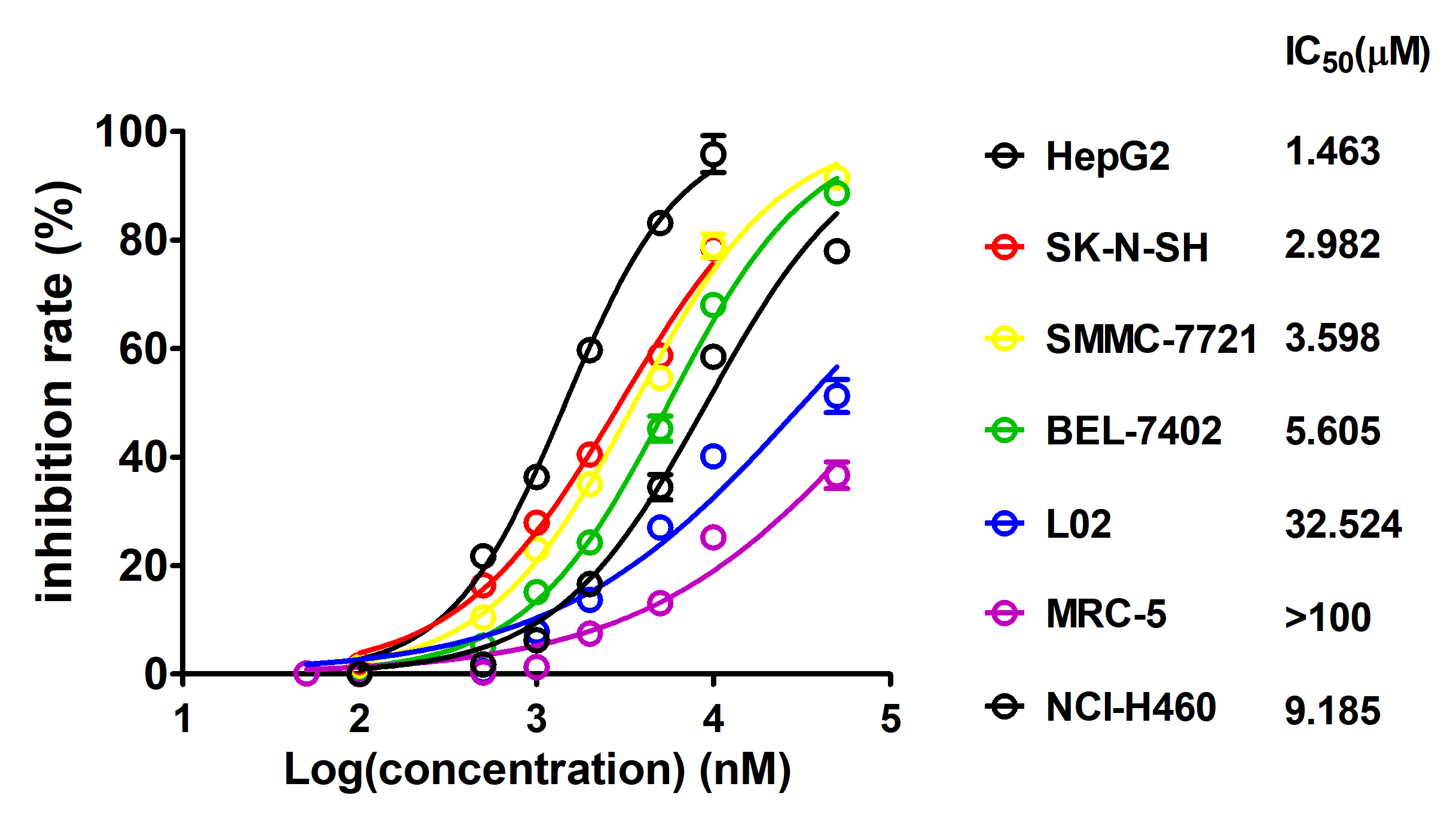


**Figure S4.** The effect of Indo5 on the growth of cells expressing TrkB alone (SMMC-7721 and SK-N-SH) or double negative cells (NCI-H460, BEL-7402 and MRC-5). The double positive cell HepG2 was used as positive control.


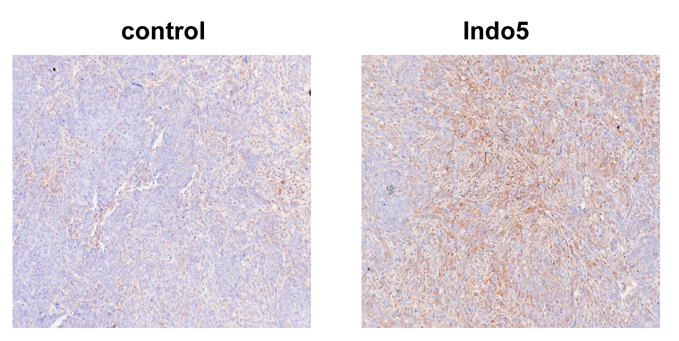


**Figure S5. TUNEL staining of HepG2 cell line tumor xenografts with Indo5 treatment.**

**
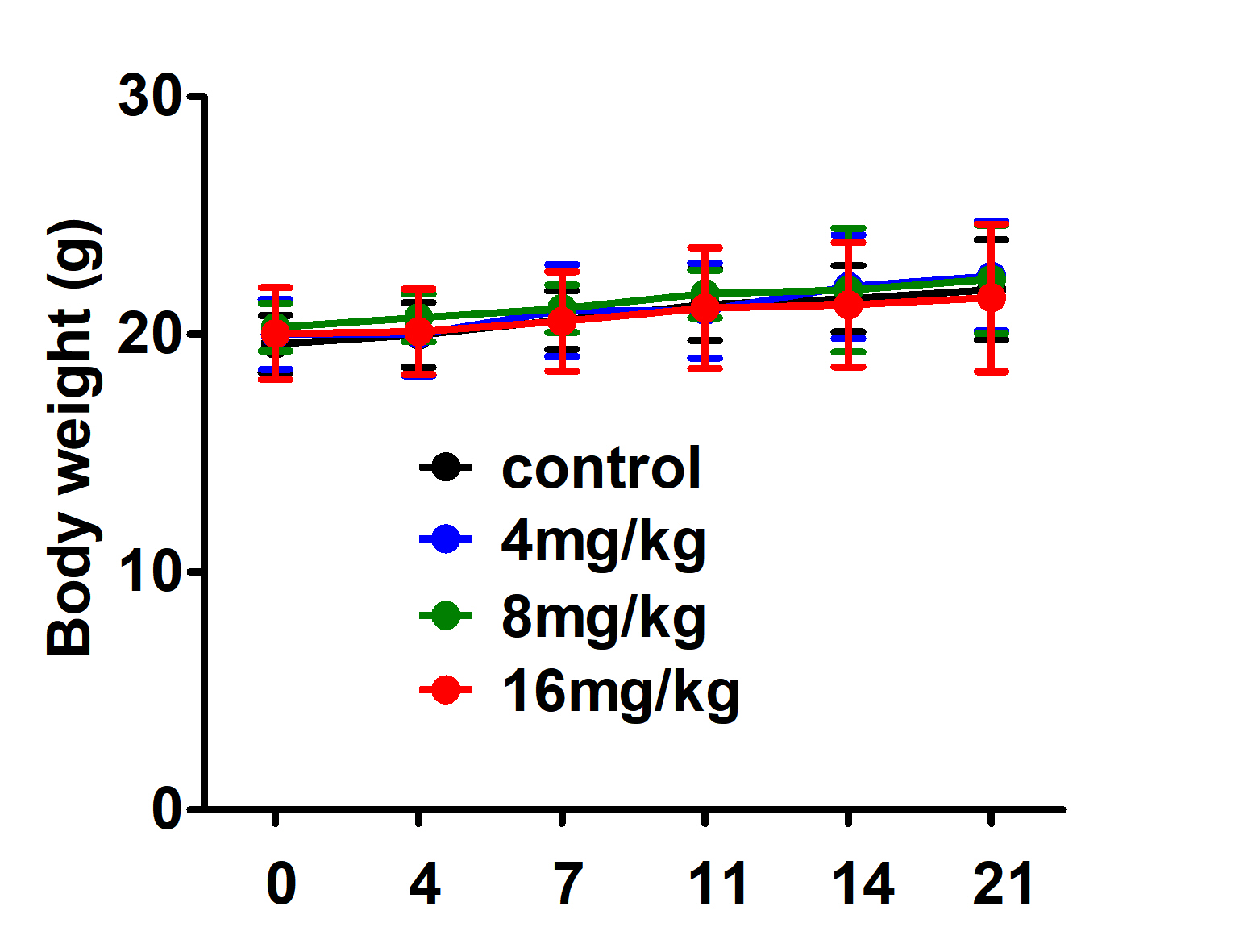
**

**Figure S6.** No obvious weight changes were observed with Indo5 treatment in HepG2 xenograft nude mice model.


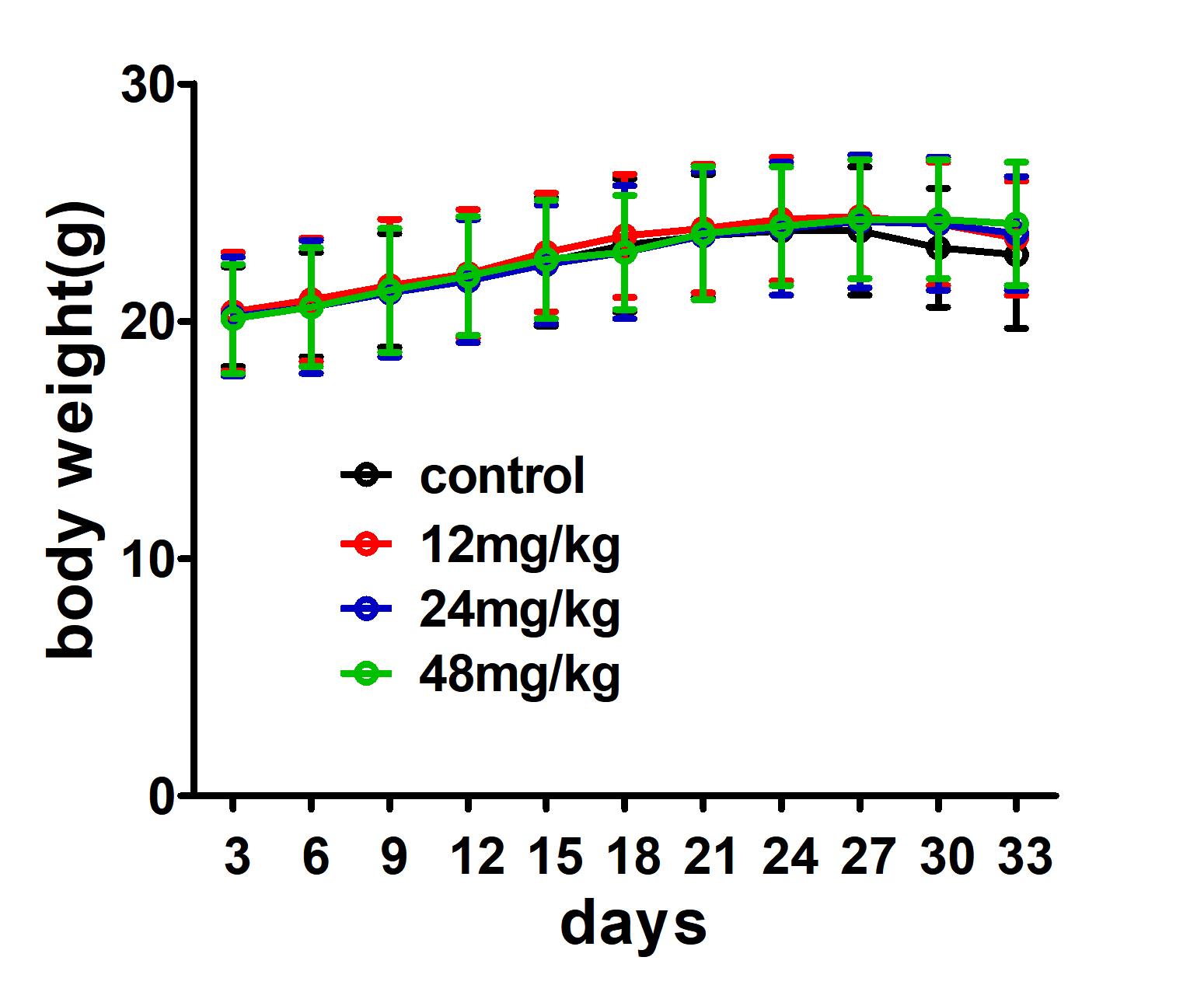


**Figure S7.** No obvious weight changes were observed with Indo5 treatment in liver orthotopic mouse model.

**
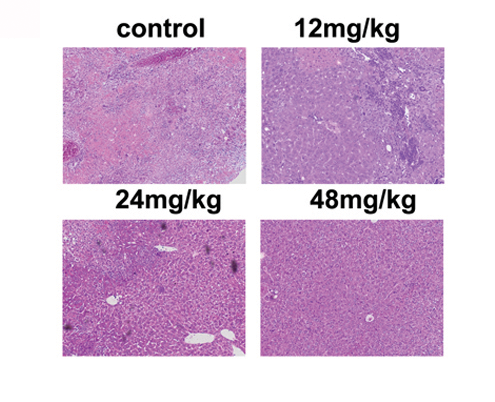
**

**Figure S8.** H&E staining of livers from mice bearing orthotopic tumors treated with the vehicle or Indo5.

**
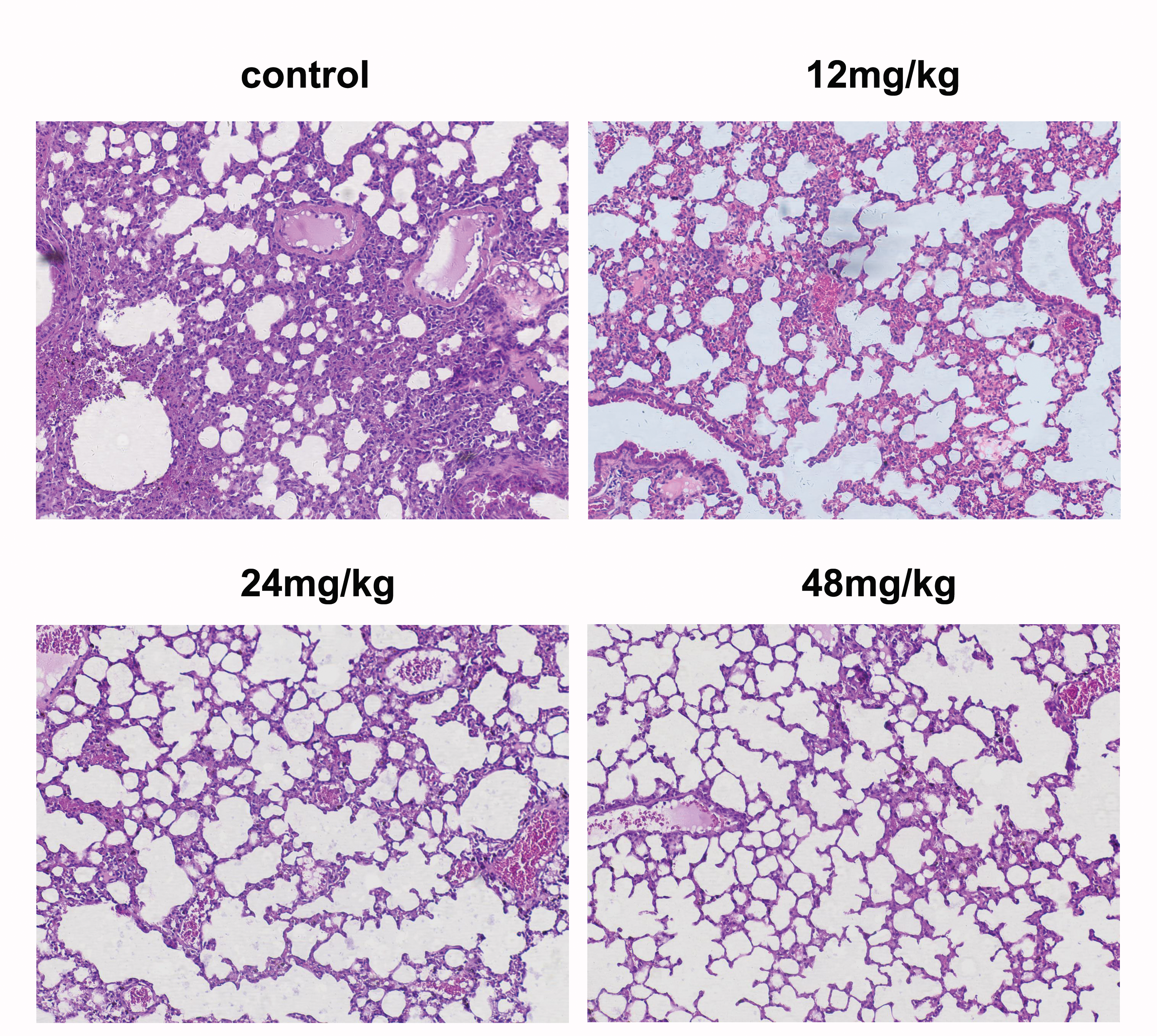
**

**Figure S9.** H&E staining of lungs from mice bearing orthotopic tumors (MHCC97H) treated with the vehicle or Indo5.


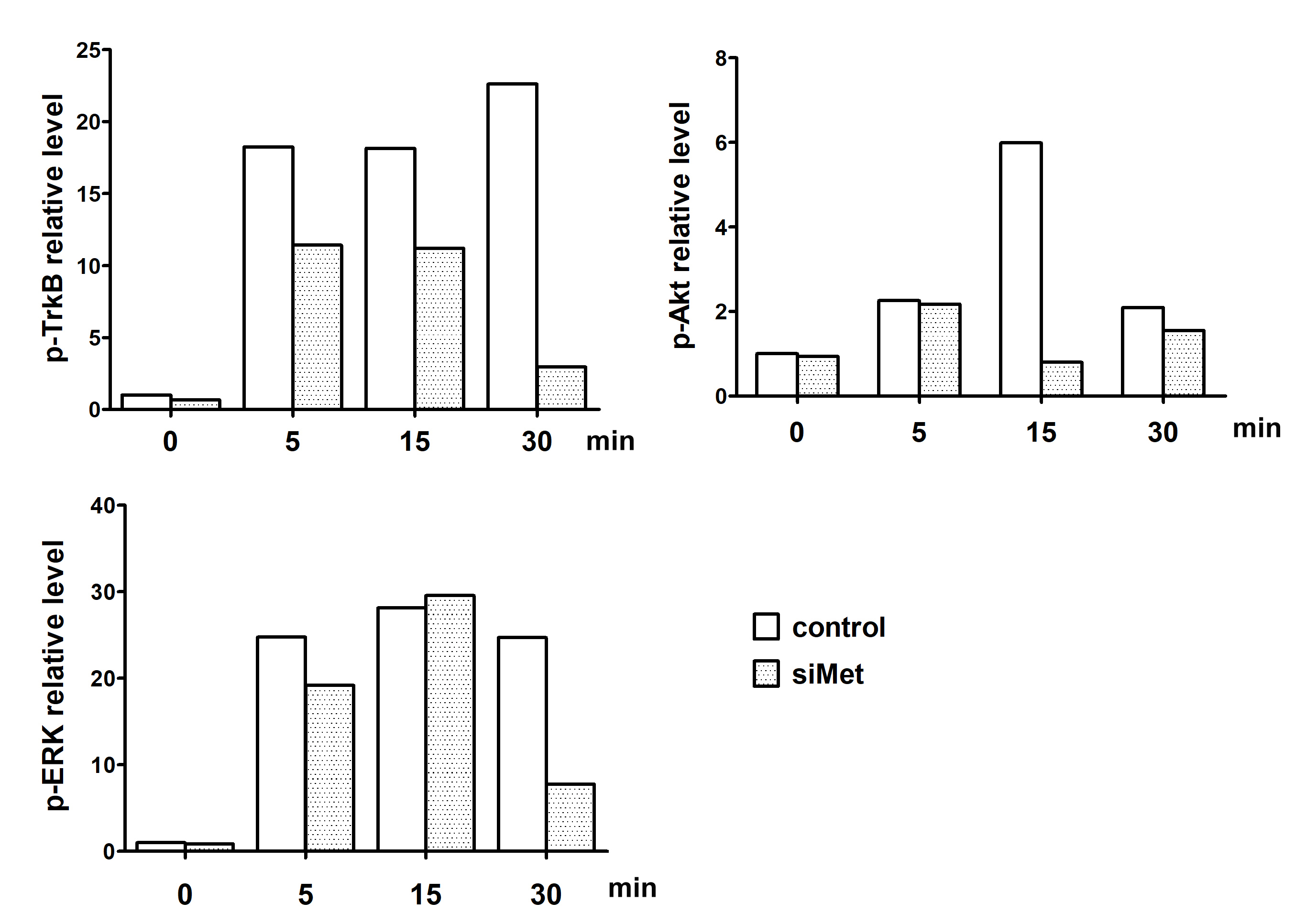


**Figure S10.** Densitometry analysis of the immunoblot bands in Fig. 5C.


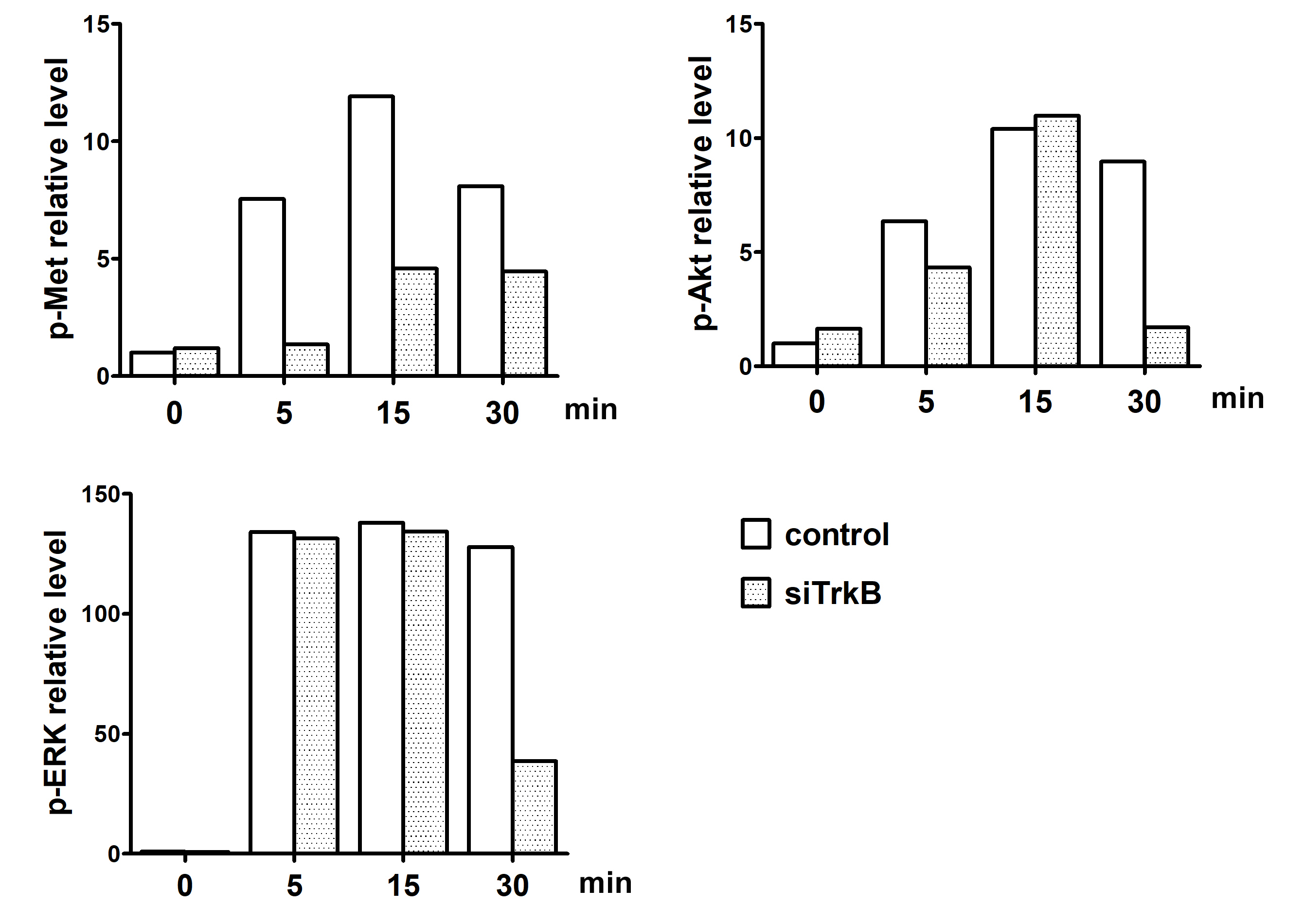


**Figure S11.** Densitometry analysis of the immunoblot bands in Fig. 5D.


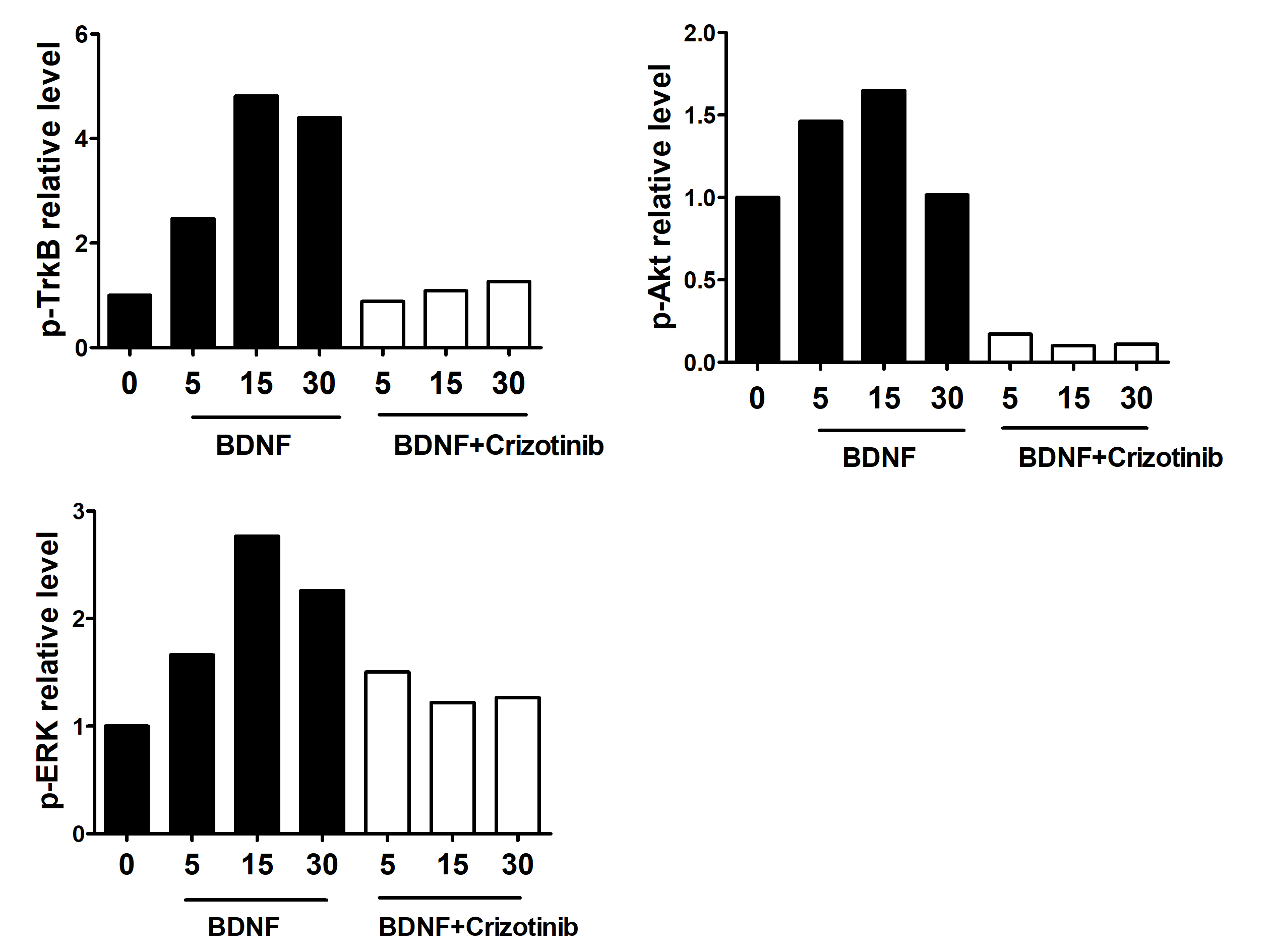


**Figure S12.** Densitometry analysis of the immunoblot bands in Fig. 5E.


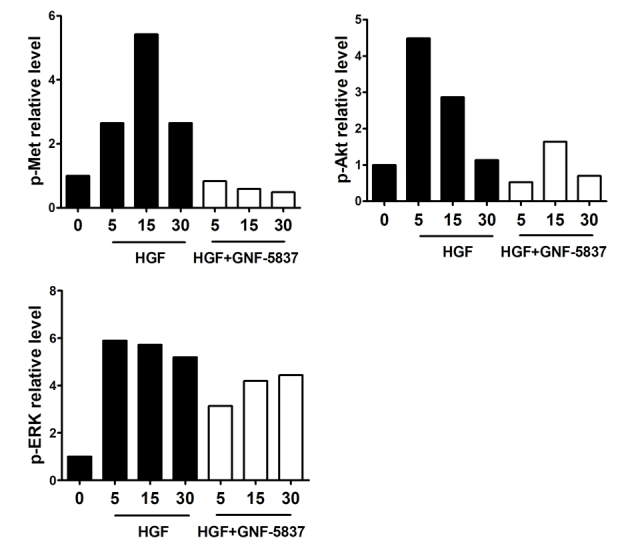


**Figure S13.** Densitometry analysis of the immunoblot bands in Fig. 5F.


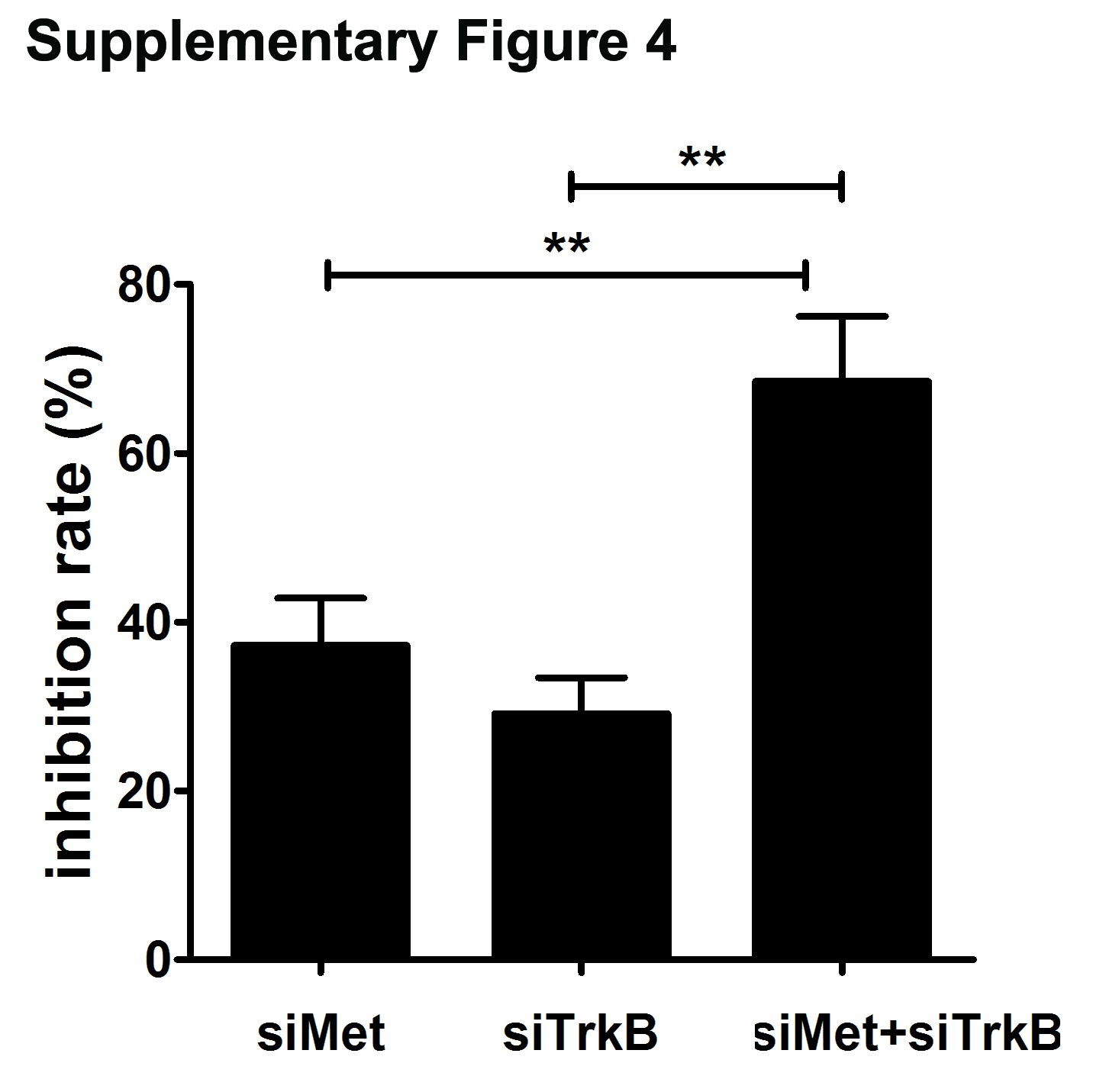


**Figure S14. The effects of c-Met and TrkB siRNA oligos on the cell growth of MHCC97H cells.** MHCC97H cells were transfected with scramble control or c-Met or TrkB siRNA oligos. Then the cell growth was measured within 72h. The inhibition rate was calculated. Data are shown as the mean ± s.d and are representative of three independent experiments. ** P < 0.01.


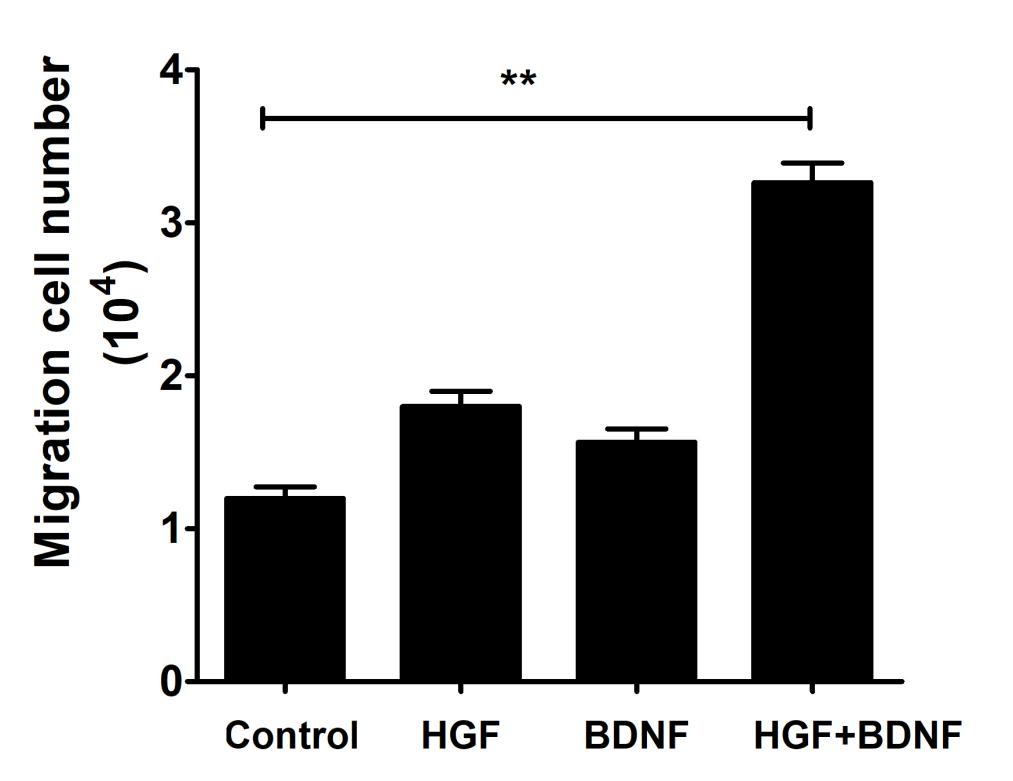


**Figure S15. HGF and BDNF co-treatment exerts synergistic promoting effects on HCC migration.** HepG2 cells were treated with HGF (5ng/ml) or BDNF (10ng/ml) as indicated and the number of cells that migrated was measured 24 h later. Data are shown as the mean ± s.d and are representative of three independent experiments. ** P < 0.01.


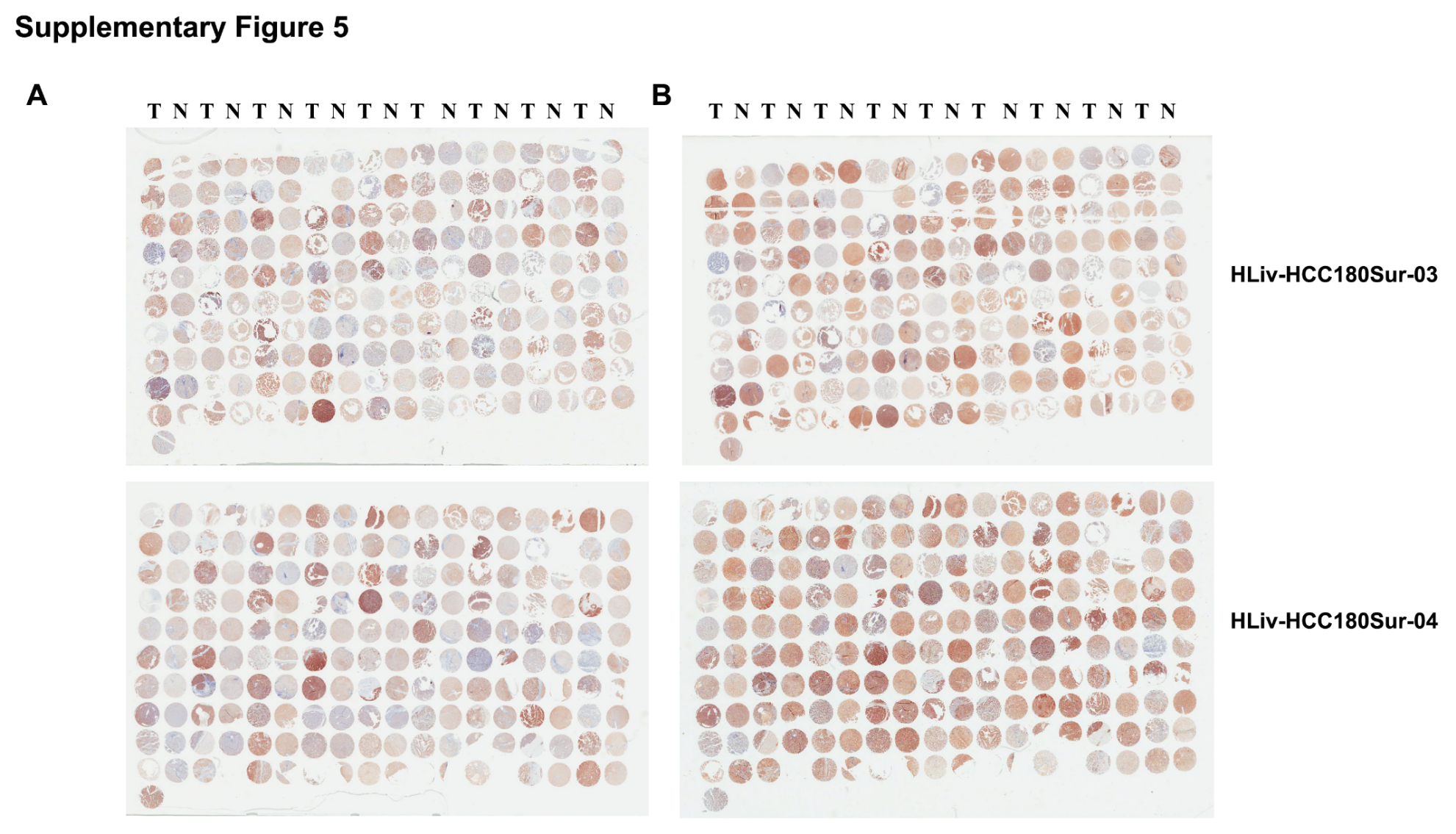


**Figure S16.** Immunohisochemical staining using specific c-Met (A) and TrkB (B) antibodies with tissue microarrays containing 180 pairs of HCC (HLiv-HCC180Sur-03, HLiv-HCC180Sur-04) and adjacent normal tissues.
